# Engineering Tfh-Specific Nanoadjuvant to Counteract Bile Acid-Induced Mitophagy and Vaccine Hyporesponsivenes

**DOI:** 10.7150/thno.125668

**Published:** 2026-03-30

**Authors:** Huimin Wu, Zhigang Zheng, Haifeng Chen, Jinchuan Liu, Xiandong Zeng, Xiaochun Huang, Xu Fang, Junlin Zhu, Yuanjia Tang, Jun Deng, Qiang Xia, Feng Xue

**Affiliations:** 1 Department of Liver Surgery and Liver Transplantation, Renji Hospital, School of Medicine, Shanghai Jiao Tong University, Shanghai, China.; 2 Shanghai Engineering Research Center of Transplantation and Immunology, Shanghai, China.; 3 Shanghai Institute of Transplantation, Shanghai, China.; 4 Department of Biliary and Pancreatic Surgery, Renji Hospital, Shanghai Jiao Tong University School of Medicine, Shanghai, China.; 5 Mini-invasive Interventional Therapy Center, Shanghai East Hospital, Tongji University, Shanghai, China.; 6 State Key Laboratory of Systems Medicine for Cancer, Shanghai Cancer Institute, Renji Hospital, Shanghai Jiao Tong University School of Medicine, Shanghai, China.; 7 Department of Obstetrics and Gynaecology, School of Clinical Medicine, LKS Faculty of Medicine, The University of Hong Kong.; 8 Shenzhen Key Laboratory of Fertility Regulation, The University of Hong Kong-Shenzhen Hospital, Shenzhen, China.; 9 Shanghai Institute of Rheumatology/Department of Rheumatology, Renji Hospital, Shanghai Jiao Tong University School of Medicine, Shanghai, China.

**Keywords:** bile acids, follicular helper T cell, mitophagy, 3-oxoLCA, nanoparticles

## Abstract

Bile acids were involved in vaccine response and modulating immune cells. Follicular helper T (Tfh) cells serve a critical immunoregulatory function in vaccine response. However, the precise role of bile acids in modulating Tfh cells and the underlying mechanisms remain unclear. Nano-adjuvant targeting and modulating Tfh cells may offer a promising strategy to improve vaccine response. In this study, we first discovered that 3-oxo-lithocholic acid (3-oxoLCA) impaired the differentiation and function of cultured Tfh vacells both in human and mice *in vitro*. *In vivo*, 3-oxoLCA gavage significantly reduced the proportion of Tfh cells in spleen and antibody responses in WT mice under NP-OVA immunization. Mechanistically, 3-oxoLCA promoted intracellular mitophagy of Tfh cells by RNA sequencing analysis and rescue experiment. Leveraging these insights, we engineered a mitophagy-inhibiting, Tfh cells-targeted nano-adjuvant (M-1@NP) decorated with anti-CXCR5 and anti-ICOS antibodies to precisely increased Tfh cells generation and function. This nanoplatform not only boosted cultured Tfh cells generation *in vitro* but also amplified germinal center (GC) responses and specific antibody production in immunized mice, with high targeting specificity and biocompatibility. In conclusion, 3-oxoLCA inhibited the differentiation and function of mouse and human Tfh cells by promoting intracellular mitophagy. Tfh cells-targeted nano-adjuvant effectively enhanced the ratio and functions of Tfh cells. M-1@NP represents a potentially valuable option for promoting vaccine response in clinical applications.

## Introduction

Vaccination remains one of the most effective public health interventions, significantly reducing the burden of infectious diseases worldwide. However, vaccine responses vary considerably among individuals, with some individuals achieving robust immunity while others exhibit poor or delayed responses. This heterogeneity poses a challenge for vaccine efficacy and highlights the need to elucidate the underlying mechanisms driving these differences. Recent researches discovered that metabolites are associated with variable vaccine responses, among which bile acids play crucial roles. Intriguingly, bile acids have also been shown to modulate immune cells, including macrophages, dendritic cells, and T cells. However, how bile acids modulate immune cells related to vaccine response remain poorly understood.

Follicular helper T (Tfh) cells, a distinct subset of effector CD4^+^ T cells, play a crucial role in vaccine responses 1. These cells are characterized by high expression levels of CXCR5, ICOS, and Bcl6, and are specifically adapted to facilitate the formation of germinal center (GC) reactions, which are instrumental in the selection of GC B cells with high-affinity antigen receptors and their subsequent differentiation into memory B cells and long-lived plasma cells 2. Recent studies have revealed that certain endogenous components can modulate Tfh cells. For instance, leptin has been shown to promote Tfh cells differentiation to enhance vaccine responses. However, whether bile acids regulate Tfh cells remains unclear.

Bile acids, the crucial component of bile, are frequently elevated in patients with cholestasis. Recently, both primary and secondary bile acids have been recognized for their potential immunomodulatory effects on immune cells 3-10. For instance, 3β-hydroxydeoxycholic acid (isoDCA) enhances Foxp3 induction by acting on dendritic cells (DCs) to diminish their immunostimulatory properties 3. CD4^+^ T effector (Teff) cells upregulate the xenobiotic transporter MDR1 (encoded by Abcb1a) in the small intestine to prevent bile acid toxicity and suppress Crohn's disease-like inflammation 4, 5. Lithocholic acid (LCA) derivatives, 3-oxoLCA and isoLCA, have been shown to inhibit Th17 cell differentiation by directly binding to the transcription factor retinoid-related orphan receptor-γt (RORγt) 6, 7. IsoalloLCA promotes Treg cells differentiation through the production of mitochondrial reactive oxygen species (mitoROS), leading to increased expression of Foxp3 via the mitoROS-CNS3-Foxp3 axis 6. LCA also metabolically induces gut RORγ^+^Helios-Treg cells 8. Additionally, oral treatment with live *Parabacteroides distasonis* (LPD) ameliorates rheumatoid arthritis pathogenesis by inhibiting Th17 cells and M2 macrophage differentiation 9. These studies collectively indicate that bile acids selectively regulate CD4^+^ T cell subsets. However, their modulatory roles, especially LCA and derivatives on Tfh and humoral immune responses in immunization remain to be clearly defined.

In the present study, we firstly discovered 3-oxoLCA inhibits the differentiation and function of human and mice Tfh cells *in vitro* and *in vivo*. Mechanistically, 3-oxoLCA modulates intracellular mitophagy in Tfh cells. Nanoparticles encapsulating mitophagy inhibitors with Tfh cells-targeting capability significantly enhanced both the Tfh cells population and antibody responses, implying targeting Tfh cells mitophagy might serve as a novel adjuvant in enhancing vaccine response.

## Methods

### Mice

The mice utilized in this investigation were aged between 6 and 12 weeks and maintained on a C57/BL6 genetic background. These animals were bred and housed under specific-pathogen-free conditions within the animal facility of Renji Hospital, Shanghai Jiao Tong University School of Medicine, where temperature was regulated to 19-23°C, humidity maintained at 45-65%, and a 12-hour dark/light cycle implemented. Wild-type (WT) C57BL/6 mice and p62-deficient (*Sqstem1*^⁻/⁻^) mutants were sourced from the Jackson Laboratory. All experimental procedures complied with the ARRIVE guidelines for animal research and were conducted in accordance with the approved institutional protocols of Shanghai Jiao Tong University.

### Vaccination and immunization

Mice received subcutaneous (s.c.) immunization in the hocks with a 100µg emulsion of NP-OVA (Biosearch Technologies) and alum (Thermo Fisher), administered either via subcutaneous injection into the hock region or through intraperitoneal (i.p.) delivery.

### Cell culture

Human naïve CD4⁺ T cells (CD4⁺CD45RA⁺CCR7⁺) were purified from peripheral blood by flow cytometry using a FACSAria III sorter (BD). The sorted cells were then seeded into 96-well plates that had been pre-coated with anti-CD3 (5 μg/mL) and anti-CD28 (1 μg/mL) antibodies for culture. The culture medium was supplemented with IL-12 (1 ng/mL, PeproTech), IL-23 (20 ng/mL, PeproTech), TGF-β (1 ng/mL, PeproTech), along with varying concentrations (0-25 μmol/mL) of the indicated bile acids (Sigma). Cells were maintained under these conditions for 3 days. Naïve CD4⁺ T cells isolated from both wild-type (WT) and *Sqstem1*^⁻/⁻^ mice were purified using a FACSAria III system (BD) and subsequently seeded into 96-well plates pre-coated with anti-CD3 (1 μg/mL) and anti-CD28 (1 μg/mL). These cells were then treated with 3-oxoLCA (0-25 μmol/L, Sigma) for a period of 3 days. To assess the effects of M-1@NP, Tfh cells were incubated for 24 h with 3-oxoLCA in the presence of either Mdivi-1 (10 μM), Blank@NP (drug-free nanoparticles, applied at an equivalent concentration), or M-1@NP (containing half the dosage of Mdivi-1).

### RNA-seq

Transcriptome sequencing was performed on mRNA extracted from naïve CD4⁺ T cells under Tfh-polarizing conditions obtained from wild-type (WT) mice, either treated with 3-oxoLCA or left untreated. RNA sequencing was conducted on the Illumina NovaSeq 6000 platform. Differential gene expression analysis was carried out using the DESeq2 package. To control the false discovery rate, p-values were adjusted following the method developed by Benjamini and Hochberg. Genes with an adjusted p-value of less than 0.05 and an absolute log₂ fold change greater than 1 were defined as statistically significantly differentially expressed.

### ELISA

The levels of NP-specific antibodies were quantified in the serum of NP-OVA-immunized mice by a sandwich ELISA assay. The following biotin-conjugated antibodies from BioLegend were used: anti-mouse IgG1 (RMG1-1, 1:500); anti-mouse IgG2c (RMG2a-62, 1:500); anti-mouse IgG2b (RMG2b-1, 1:500); and anti-mouse IgG3 (RMG3-1, 1:250). IL-21 levels in culture supernatants were measured using ELISA kits (MultiSciences) according to the manufacturer's protocols, for both mouse and human naïve CD4⁺ T cells.

### Flow cytometry

Single-cell suspensions were generated from splenic tissue and labeled with a panel of fluorescently conjugated monoclonal antibodies. The specific antibodies used included: for human samples anti-B220-AF700, anti-GL-7-FITC, anti-Fas-PE-Cy7, anti-CD138-BV711, anti-IgD-APC/Cy7, anti-CD44-PE/Cy7, anti-CD62L-BV711, anti-TACI-PE, anti-PD-1-BV421, anti-IgG1-APC, anti-IFN-γ-PE-Cy7, anti-IL-4-PE, and anti-CD19-FITC; for murine samples—anti-CCR7-APC/Cy7, anti-CD45RA-PE-Cy7, anti-CXCR5-APC, and anti-PD-1-BV711. Human-targeted antibodies and Alexa Fluor 647 Streptavidin were acquired from BioLegend. Additional reagents including anti-IgM-BV421, anti-CD38-FITC, anti-Bcl6-PE, and anti-CD4-BV510 were obtained from BD Pharmingen. Anti-IL-21-PE, anti-Foxp3-FITC, and anti-human IL-21-PE were sourced from eBioscience, while NP-BSA-Biotin was procured from Biosearch Technologies. For the detection of intracellular cytokines, cells were first activated with PMA and ionomycin for 5 h in the presence of monensin or brefeldin A, and then processed for staining. Subsequent intracellular staining was carried out using the Fixation/Permeabilization Solution Kit (BD Pharmingen). Transcription factors Bcl6 and Foxp3 were stained with the Foxp3/Transcription Factor Staining Buffer Set (eBioscience). Cell viability was assessed using 7-AAD (7-Aminoactinomycin D; Thermo Fisher), propidium iodide (PI; BioLegend), or the Zombie Aqua™ Fixable Viability Kit (BioLegend) to exclude non-viable cells.

### Western blot

Cultured naïve CD4⁺ T cells from both human and mouse origins were lysed using RIPA buffer (Sigma-Aldrich) containing a protease inhibitor cocktail (Roche Diagnostics). Protein concentration was quantified with the Pierce BCA Protein Assay Kit (Thermo Fisher Scientific). Equal amounts of protein samples were separated electrophoretically on 8% SDS-polyacrylamide gels and then transferred onto PVDF membranes (MilliporeSigma). The membranes were blocked with 5% skim milk in PBST (PBS containing 0.1% Tween-20) for one hour at room temperature before being probed with the following primary antibodies: anti-LC3II (1:1000), anti-p62 (1:1000), anti-Pink1 (1:1000), and anti-β-actin (1:5000) from Cell Signalling Technology; anti-Bcl6 (1:500) was acquired from BioLegend. Subsequently, membranes were incubated with horseradish peroxidase (HRP)-conjugated anti-rabbit IgG secondary antibodies, and protein bands were visualized using chemiluminescent substrates (GE Healthcare Biosciences).

### Real-time quantitative PCR

Total RNA was extracted from cultured cells or tissue samples using TRIzol® reagent (Invitrogen). Subsequently, complementary DNA (cDNA) was synthesized from 1 µg of total RNA using a commercial cDNA Synthesis Kit (Takara), which employs an optimized blend of reverse transcriptase and random hexamer/primer oligo(dT) mixtures to ensure high-efficiency first-strand synthesis. Quantitative real-time PCR (qPCR) was performed using SYBR® Green Master Mix. Each reaction was run in triplicate under the following cycling conditions: initial denaturation at 95 °C for 10 min, followed by 40 cycles of 95 °C for 15 s and 60 °C for 1 min. Gene-specific PCR primers were designed to span exon-exon junctions where applicable to avoid genomic DNA amplification. All mouse-specific primer sequences are provided in Supplementary Table 1, while human-specific primers can be found in Supplementary Table 2. The relative expression of each gene of interest was normalized to the endogenous reference gene β-actin using the comparative threshold cycle (CT) method. Briefly, the ΔCT value was calculated for each sample by subtracting the CT value of β-actin from that of the target gene. Fold changes in expression were then determined relative to a calibrator sample (such as a control group).

### Transmission electron microscopy

To examine morphological alterations in T cells, naïve CD4⁺ T cells were isolated from the spleens of wild-type (WT) mice and cultured at a density of 2 × 10⁶ cells per well under Tfh-polarizing conditions. The culture was maintained in complete RPMI-1640 medium devoid of 2-mercaptoethanol, supplemented with 10% fetal bovine serum (FBS), 1 mM sodium pyruvate, 10 mM HEPES, and 1× non-essential amino acids. For primary fixation, the cells were treated overnight at 4 °C with a mixture of 2.5% glutaraldehyde and 4% paraformaldehyde in PBS. After subsequent steps of dehydration, embedding, and staining, the prepared samples were visualized using a transmission electron microscope (JEOL, Japan). Mitochondria were delineated through a combined approach of manual and automated segmentation. The cross-sectional area of individual mitochondria was quantified based on pixel measurements derived from TEM images, using ImageJ software (version 1.52, Bethesda).

### Preparation of M-1@NP

The nanoparticles were fabricated via a double emulsion solvent evaporation technique. Specifically, 5 mg of lipophilic Mdivi-1 and 2.5 mg of DSPE-PEG₂₀₀₀-Mal were solubilized in 1 mL of chloroform. This organic phase was then emulsified by sonication at 300 W in an ice bath for 3 minutes. Subsequently, 5 mL of an aqueous solution containing 1% (v/v) F68 was introduced, and the mixture was further sonicated under the same conditions. The resulting nanoparticles were collected via centrifugation at 15,000 rpm for 40 minutes, and the pellet was resuspended in PBS. For antibody conjugation, a thiol-maleimide coupling strategy was employed. Prior to conjugation, 30 μL each of anti-ICOS and anti-CXCR5 antibodies (100 μg/mL) were reduced with 30 μL of TCEP (0.5 M) at room temperature for 15 minutes. The activated antibodies were promptly introduced into 2 mL of nanoparticle suspension (1 mg/mL) and allowed to react under continuous stirring at 4 °C overnight. The conjugated nanoparticles were purified through three washing cycles and stored at 4 °C in chilled PBS. The encapsulation efficiency of Mdivi-1 was quantified by analyzing the amount of unentrapped drug in the supernatant using high-performance liquid chromatography (HPLC). Meanwhile, the conjugation efficiency of the anti-ICOS and anti-CXCR5 antibodies was assessed with a BCA protein assay.

### Characterization of M-1@NP

The morphology of the nanoparticles was characterized using transmission electron microscopy (TEM, FEI Talos F200X). The size distribution and zeta potential of nanoparticles were measured with Nanosizer (Nano ZS90, Malvern) to assess size uniformity and surface charge. Surface modification of the antibodies was evaluated using nuclear magnetic resonance (NMR; Bruker 600 MHz, Germany) and X-ray photoelectron spectroscopy (XPS; Thermo Scientific K-Alpha, USA). The state of Mdivi-1 encapsulated within the nanoparticles was analyzed using X-ray diffraction (XRD; Rigaku Ultima IV, Japan) to determine its crystalline or amorphous nature. Fourier-transform infrared spectroscopy (FTIR; Thermo Fisher Scientific Nicolet iS20, USA) was employed to investigate potential chemical changes during nanoparticle preparation and to evaluate conjugation efficiency. The stability of the nanoparticles was assessed by monitoring changes in particle size over time in PBS containing varying concentrations of fetal bovine serum.

### Confocal microscope

Tfh cells were incubated for 24 h with either free rhodamine dye or rhodamine-conjugated M-1@NP. Following incubation, cells were extensively washed, fixed, and counterstained with DAPI for fluorescence microscopy.

### Screening for the optimal targeting ratio of anti-ICOS and anti-CXCR5

Based on the previously described nanoparticle preparation protocol, Cy7-labeled M-1@NP conjugated with anti-CXCR5 and anti-ICOS antibodies at varying molar ratios (anti-CXCR5: anti-ICOS = 1:1, 1:0.5, or 1:0.2) were synthesized. These functionalized nanoparticles were then co-incubated with Tfh cells at 37 °C for 24 h. After incubation, the Tfh cells were washed three times and subsequently analyzed by flow cytometry.

### Encapsulation rate and drug loading rate

The encapsulation efficiency (EE) and drug loading (DL) of the nanoparticles were determined using high-performance liquid chromatography (HPLC; Agilent 2000, Germany).

### Drug releasing ability of M-1@NP *in vivo*

To evaluate the release kinetics of Mdivi-1 from M-1@NP nanoparticles, samples of 1 mL each containing either free Mdivi-1 or M-1@NP were collected from a dialysis bag (MWCO = 10,000 Da; Shanghai Greenbird Biotechnology Co., Ltd.) at 24-hour intervals over a period of seven days. After each withdrawal, an equivalent volume of fresh PBS was introduced to maintain constant volume conditions. The concentration of Mdivi-1 in the collected samples was quantified using high-performance liquid chromatography (HPLC) on an Agilent 2000 system (Germany).

### Biodistribution of M-1@NP *in vivo*

Male C57BL/6 mice received an intravenous injection via the tail vein of 200 μg of Cy7-labeled nanoparticles, which were either Uncoupled Ab nanoparticles (lacking conjugated anti-ICOS and anti-CXCR5 antibodies) or M-1@NP. Four h post-injection, the *in vivo* distribution of nanoparticles across various organs was monitored using a fluorescence imaging system (LB983, Berthold Technologies GmbH & Co., KG, Bad Wildbad, Germany). Subsequently, major organs—including the heart, liver, spleen, lungs, kidneys, and left axillary lymph nodes—were excised and subjected to *ex vivo* fluorescence imaging with the same equipment.

### *In vivo* pharmacokinetics

Male C57BL/6 mice were injected with free Mdivi-1 or M-1@NP (Mdivi-1, 25 mg/kg) via the tail vein. At each designated time point, 200 μL of whole blood was collected from mice. The concentration of Mdivi-1 in these samples was subsequently quantified by high-performance liquid chromatography (Agilent 2000, Germany).

### Biocompatibility

Primary splenocytes were extracted and cultured in 96-well plates, followed by the addition of varying concentrations of M-1@NP. After 48 h, the nanoparticle supernatant was discarded, and cell viability was measured using the CCK-8 assay according to the manufacturer's instructions. *In vitro* biocompatibility assessment. Eight-week-old male C57BL/6 mice received intravenous injections every two days over one week with PBS, Mdivi-1, Black@NP, or M-1@NP (equivalent to 25 mg/kg of Mdivi-1). On day 7, the mice were sacrificed, and major organs were collected for histopathological examination. Blood samples were also collected for biochemical analysis.

### Hematoxylin-eosin (H&E) staining

Organ slices embedded in paraffin underwent deparaffinization using xylene and were subsequently rehydrated through an ascending ethanol gradient. These sections were next subjected to hematoxylin staining for a duration of 5 minutes, after which they were differentiated in an alcohol solution containing 0.1% hydrochloric acid. This was followed by eosin staining, which lasted 2 minutes, and then dehydration via a descending ethanol series. Once cleared in xylene, the samples were coverslipped with a neutral mounting medium. Finally, the prepared sections were examined and imaged under a microscope.

### Statistical analysis

Immunized WT mice treated with or without 3-oxoLCA, and naïve CD4^+^ T cells with or without 3-oxoLCA treatment were analyzed by non-parametric two-sided Mann-Whitney U-tests. The effects of different doses of 3-oxoLCA on both human and mouse Tfh cell cultures were examined, and the resulting antibody titers in mice were analyzed using one-way or two-way ANOVA, respectively. *P* value <0.05 was considered statistically significant. GraphPad Prism (version 9.0) were used to analyze the data.

## Results

### 3-oxoLCA inhibits human Tfh cell differentiation and function

**Figure 1:**
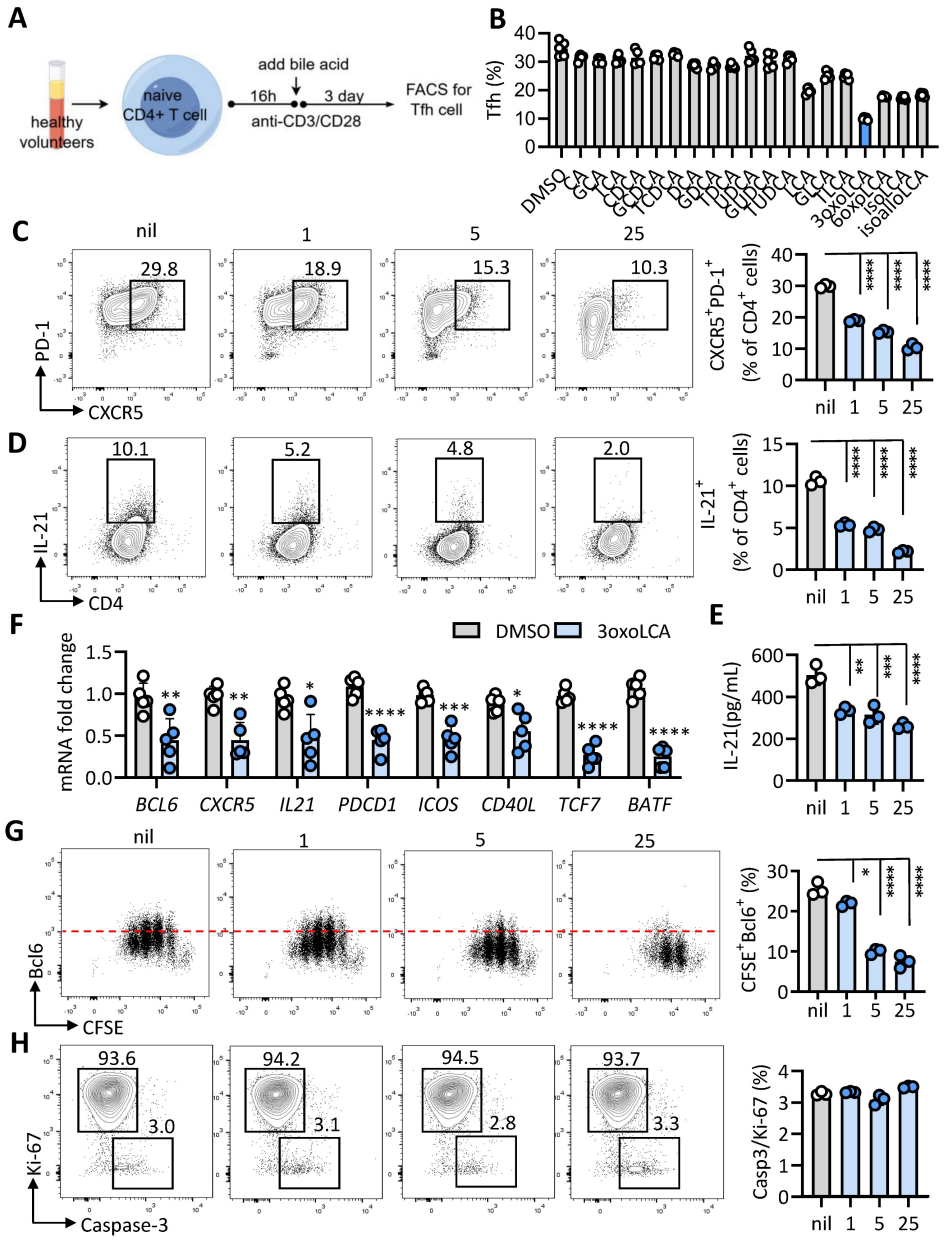
** 3-oxoLCA inhibits human Tfh cells differentiation.** A. Schematic of human CD4^+^ T cell culture, bile acids treatment and assay. B. The percentages of CD4^+^CXCR5^+^PD-1^+^ Tfh cells from cultured CD4^+^CD25^-^CD45RA^+^CCR7^+^ naive CD4^+^ T cells isolated from PBMCs of healthy volunteers stimulated with anti-CD3/CD28, and further treated with various bile acids (25 μM) for 3days (n=5). C-E. Representative FACS plots and statistics showing the percentage of CXCR5^+^PD-1^+^ Tfh cells (C), and ratio (D) and concentration (E) of IL-21 in cultured human naive CD4^+^CD25^-^CD45RA^+^CCR7^+^ cells with 3-oxoLCA treatment for 3days (n=3). F. Real-time quantitative PCR was employed to assess genes related to Tfh cells in human naïve CD4⁺ T cells after 12h of anti-CD3/CD28 activation followed by 12h of 3-oxoLCA treatment (n=5). G-H. CFSE-labelled proliferation assay of Bcl6 expression (G), and apoptotic analysis (H) of cultured human naive CD4^+^CD25^-^CD45RA^+^CCR7^+^ cells with 3-oxoLCA treatment for 3days (n=3). The graphs show the data as mean ± SEM. *p < 0.05, **p < 0.01, ***p < 0.001, ****p < 0.0001.

The bile acid pool is composed of both primary and secondary bile acids. Primary bile acids, such as cholic acid (CA) and chenodeoxycholic acid (CDCA), are synthesized in hepatocytes and subsequently stored in the gallbladder. In contrast, secondary bile acids—including deoxycholic acid (DCA), lithocholic acid (LCA), and ursodeoxycholic acid (UDCA)—are generated through bacterial metabolism 11, 12. To screen which bile acids potentially modulate Tfh cells generation, human naïve CD4^+^ T (CD4^+^CD25^-^CD45RA^+^CCR7^+^) cells were isolated from healthy volunteers, and stimulated with anti-CD3 and anti-CD28 for 16 h, followed by CA, CDCA, DCA, UDCA, LCA and 3-oxoLCA, isoLCA, isoalloLCA, alloLCA and 6-oxoLCA treatment for additional for 3 days at concentration of 25μM (Figure 1A). Notably, we found that 3-oxoLCA significantly decreased the proportion of PD-1^+^CXCR5^+^ Tfh cells (Figure 1B). Therefore, we focused on the 3-oxoLCA in regulating Tfh cells. We further observed that 3-oxoLCA restrained Tfh cells in a dose-dependent manner (Figure 1C), which in line with previous studies that 3-oxoLCA exerts modulating effects on Th17 cells under 5 μM 6. Moreover, we observed that 3-oxoLCA restricted the production of IL-21 in a dose-dependent manner by FACS and ELISA assay (Figure 1D, F). The mRNA levels of Tfh signature genes, such as *BCL6, PDCD1, ICOS, CD40L,* and* IL21* were significantly decreased (Figure 1E), as well as the Bcl6 protein expression Figure S1B). We analyzed the impacts of 3-oxoLCA on Tfh cells proliferation (CFSE and Ki-67) and apoptosis (active caspase 3). Results shown that 3-oxoLCA dramatically inhibited proliferative Tfh cell signature markers CFSE^+^Bcl6^+^, CFSE^+^CXCR5^+^, CFSE^+^IL-21^+^ cells as low as 5 μM, displaying in a dose-dependent inhibition manner (Figure 1G, S1C). We next tested the expression of proliferation marker (Ki-67) and apoptosis maker (active caspase 3) upon 3-oxoLCA treatment, results shown that there were no differences on Ki-67, Caspase 3 expression and the Caspase 3/Ki-67 ratio (Figure 1H). These results demonstrated that 3-oxoLCA restrained the differentiation and function of human Tfh cells *in vitro*. Taken together, 3-oxoLCA impaired the differentiation and IL-21 secretion of human Tfh cells *in vitro*.

### 3-oxoLCA impairs mouse Tfh cells and germinal center responses *in vivo*

**Figure 2:**
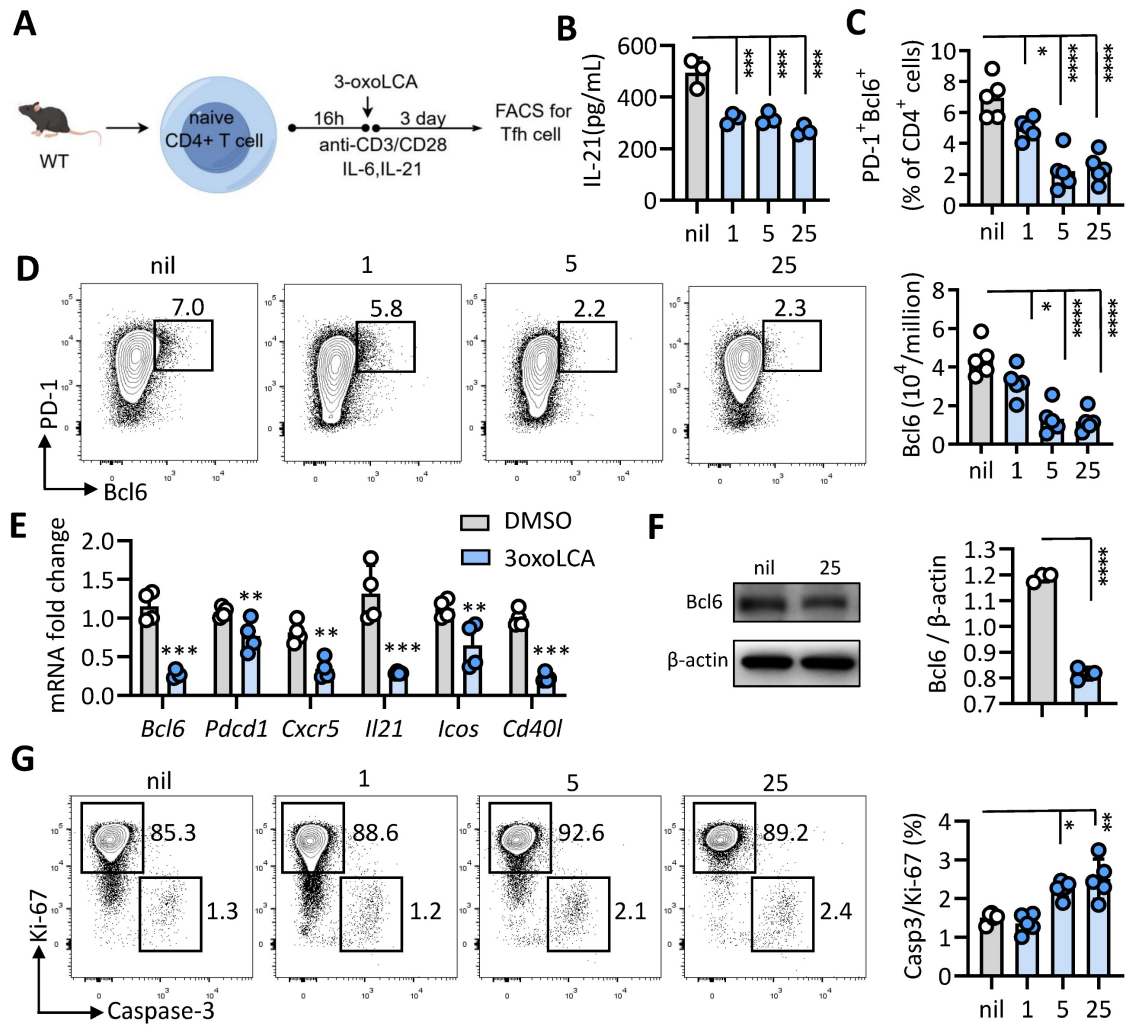
** 3-oxoLCA suppresses mouse Tfh cells differentiation.** A. Workflow of the experimental procedure of mouse CD4^+^CD25^-^CD44^-^CD62L^+^ naïve T cells from wild-type C57BL/6 mice, activation with anti-CD3/CD28 and 3-oxoLCA treatment. B. IL-21 levels in cultured mouse naïve CD4^+^ T cells treated with or without 3-oxoLCA for 2 days by ELISA (n=3). C-D. Representative FACS plots showing the differentiation of the percentage and numbers of Bcl6^+^PD-1^+^ Tfh cells from cultured naïve CD4^+^ T cells (n=5). E. Real-time quantitative PCR was performed to measure Tfh-related genes expression in mouse naïve CD4⁺ T cells after 12 h of anti-CD3/CD28 activation, followed by a 12h treatment with 3-oxoLCA (n=5). F. Bcl6 expression in mouse naïve CD4^+^ T cells treated with or without 3-oxoLCA for 24h by Western blot analysis (n=3). G. Representative FACS plots showing the proliferation (Ki-67), and apoptosis (active caspase 3) in cultured mouse CD4^+^ T cells treated with 3-oxoLCA for 3 days (n=5). The graphs show the data as mean ± SEM. *p < 0.05, **p < 0.01, ***p < 0.001, ****p < 0.0001.

Given its effect elsewhere, we extended our inquiry to determine if 3-oxoLCA also suppresses the differentiation of mouse Tfh cells. Naïve CD4⁺ T cells isolated from WT C57BL/6 mice were stimulated with anti-CD3/CD28 and were either treated with 25 μM 3-oxoLCA or left untreated, as shown in Figure 2A. Consistent with its effects on human Tfh cells, 3-oxoLCA treatment potently reduced IL-21 secretion from mouse Tfh cells, as measured by ELISA (Figure 2B). Furthermore, FACS analysis revealed that the treatment dose-dependently suppressed the proportion and number of Bcl6^+^PD-1^+^ Tfh cells, as well as Bcl6 expression levels (Figures 2C, 2D, and S2A). Following a 12-hour treatment with 25 µM 3-oxoLCA, the transcript levels of key Tfh cells-related genes (including *Bcl6*, *Pdcd1*, *Icos*, *Cd40l*, *Il21* and *Cxcr5*) were remarkably downregulated. (Figure 2E). Also, western blot analysis revealed that the protein level of Bcl6 was also downregulated by 3-oxoLCA treatment (Figure 2F). At doses of 5 µM and 25 µM, 3-oxoLCA treatment elevated the Caspase 3/Ki-67 ratio, indicating that higher concentrations promote apoptosis in mouse Tfh cells (Figure 2G, S2B). Collectively, these results establish that 3-oxoLCA suppresses the *in vitro* generation of both human and mouse Tfh cells, along with their production of IL-21.

**Figure 3:**
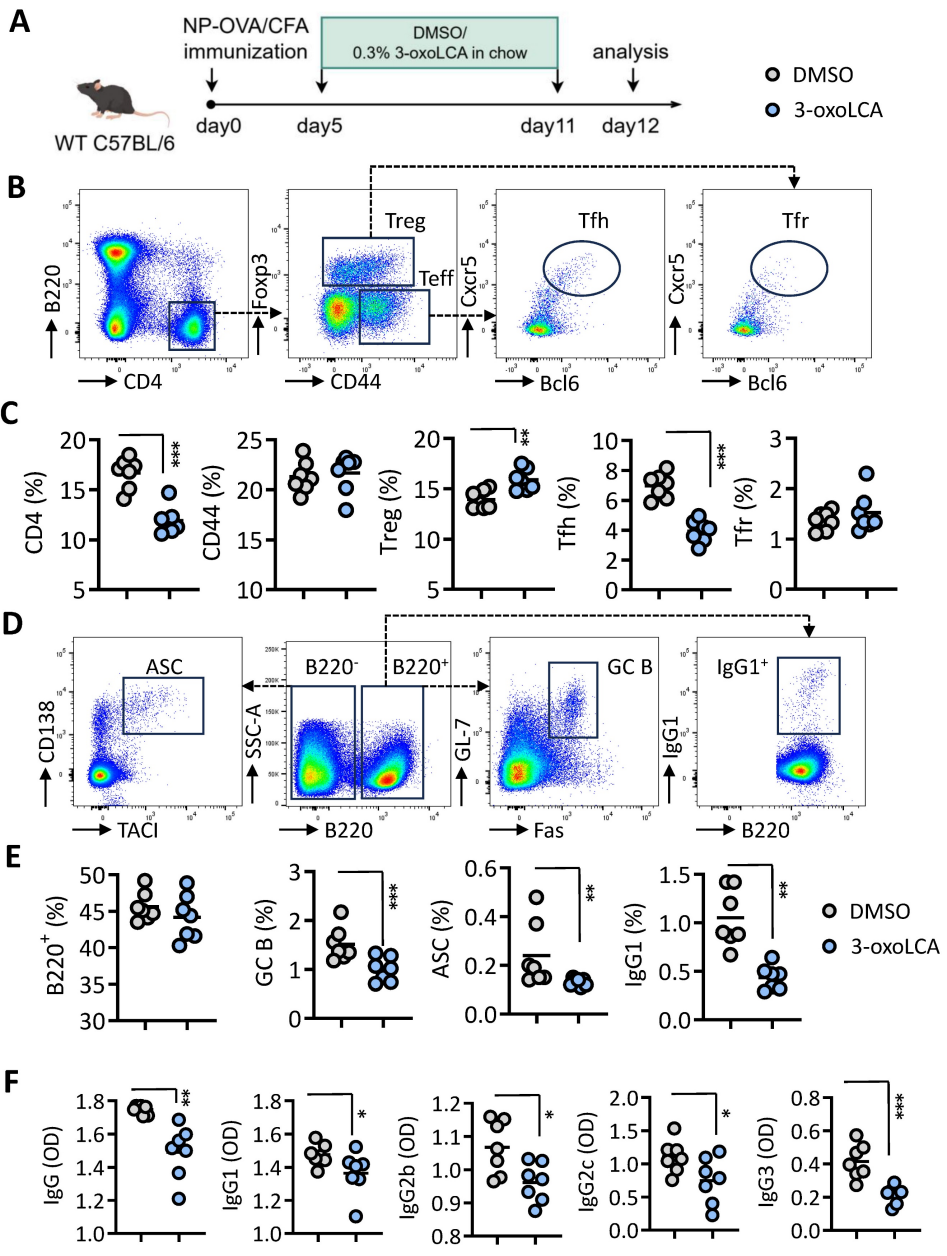
** 3-oxoLCA dampens mouse Tfh cells and humoral immune response *in vivo*.** A. Workflow of the experimental procedure of WT C57BL/6 mice with NP-OVA/CFA immunization (subcutaneous), 3-oxoLCA (oral gavage) and analysis. B-E. Representative FACS plots of ZA^-^B220^-^CD4^+^CD44^+^Foxp3^-^CXCR5^+^Bcl6^+^ Tfh cells, ZA^-^B220^-^CD4^+^CD44^-^Foxp3^+^CXCR5^+^Bcl6^+^ Tfr cells, CD44^-^Foxp3^+^ Treg cells and Foxp3^-^CD44^+^ effector cells (B), along with ratio statistics (C); as well as ZA^-^B220^-^CD138^+^TACI^+^ antibody secreting cells (ASC), ZA^-^B220^+^GL-7^+^Fas^+^ germinal center (GC) B cells, and ZA^-^B220^+^IgG1^+^ cells (D), and ratio statistics (E) in the spleens of WT C57BL/6 mice with NP-OVA/CFA immunization, treated with or without 3-oxoLCA (n=7). F. ELISA assay for serum NP-specific IgG1, IgG2b, IgG2c and IgG3 titers from in WT C57BL/6 mice immunized with NP-OVA/CFA, followed by 3-oxoLCA treatment (n=7). The graphs show the data as mean ± SEM. *p < 0.05, **p < 0.01, ***p < 0.001, ****p < 0.0001.

Then, we examined the effect of 3-oxoLCA in the quantity and function of Tfh cells and antibody response in immunized mice. To this end, mice were immunized subcutaneously via an emulsion of 4-hydroxy-3-nitrophenyl (NP)-conjugated ovalbumin (OVA) using complete Freund's adjuvant (CFA). After 5 days, mice were intragastrically administered with DMSO or 3-oxoLCA (0.3%) every other day from day 5 to day 11. On day 12, mice were harvest for flow cytometric analysis of Tfh cells and B cells in spleen (Figure 3A). Results shown that the percentages of CD4^+^ T cells, CD4^+^Foxp3^+^ Treg cells, as well as CD4^+^Foxp3^-^CD44^+^CXCR5^+^Bcl6^+^ Tfh cells in spleen were all notably reduced in mice treated with 3-oxoLCA compared to control mice. However, the percentages of T effector cells (CD4^+^CD44^+^) and Tfr cells (CD4^+^Foxp3^+^CD44^-^CXCR5^+^Bcl6^+^) were comparable between 3-oxoLCA-treated mice and control mice (Figure 3B,C). Likewise, there were significant decrease of the percentage and numbers of GC B cells (GL-7^+^Fas^+^), ASCs (CD138^+^TACI^+^) and IgG1^+^ B cells of mice with 3-oxoLCA treatment in comparison to DMSO-treated mice (Figure 3D, E). However, the percentages of total B220^+^ cells remain unchanged. ELISA assay confirmed that serum anti-NP IgG, IgG1, IgG2b, IgG2c and IgG3 were significantly declined in mice with 3-oxoLCA treatment compared to control mice (Figure 3F). Collectively, these results suggest that 3-oxoLCA impairs mouse Tfh cells differentiation and antibody responses *in vitro* and *in vivo*.

### 3-oxoLCA induces mitophagy in Tfh cells

**Figure 4:**
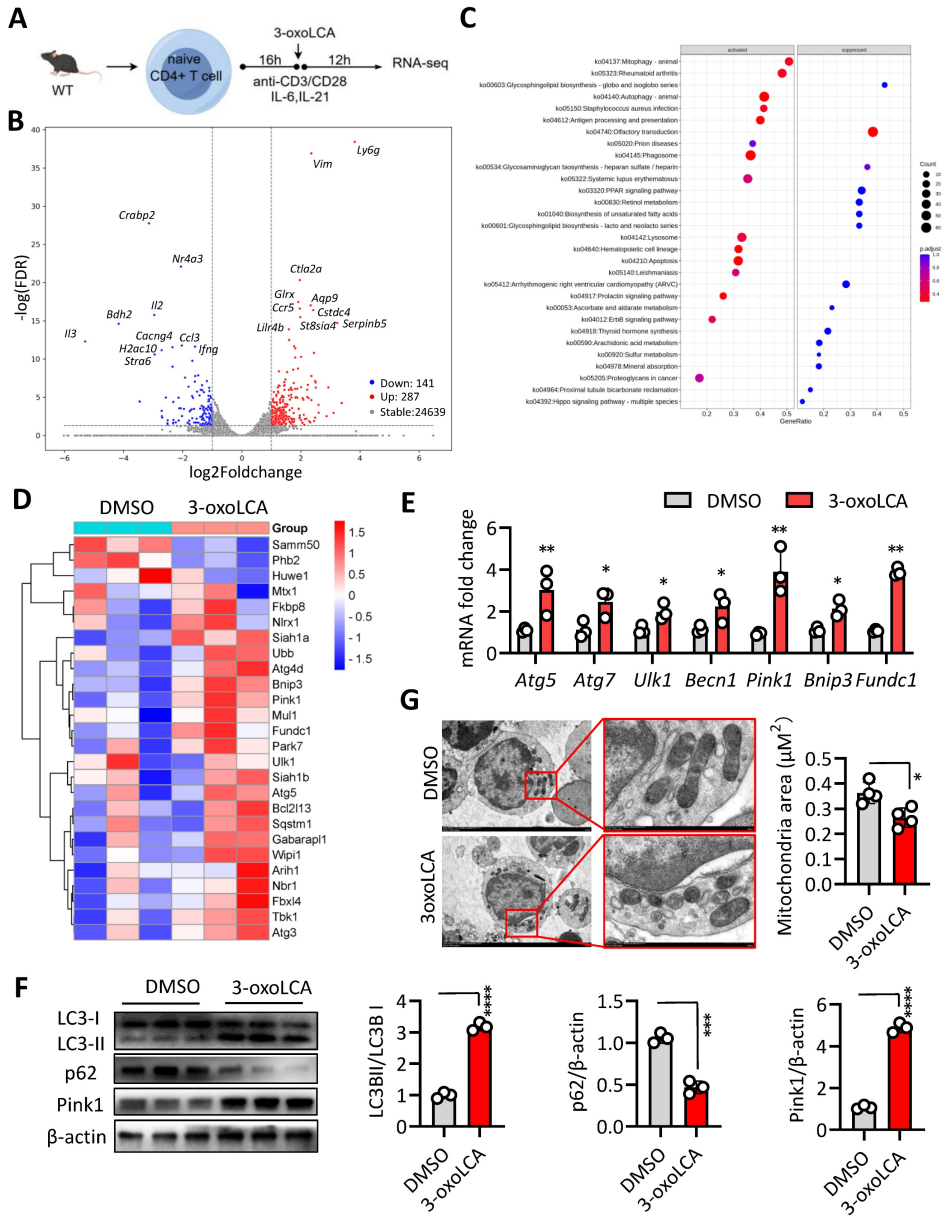
** 3-oxoLCA induces mitophagy in mouse Tfh cells.** A. Experiment design of mouse Tfh cells culture, 3-oxoLCA treatment and RNA-seq analysis. B-D. Volcano plot showing differentially expressed genes (DEGs) (B), gene set enrichment analysis (GSEA) (C), and heatmap for mitophagy related genes in cultured mouse CD4^+^ T cells, 3-oxoLCA treatment and RNA-seq analysis. E. Real-time quantitative PCR analysis for mitophagy-related genes in cultured mouse CD4^+^ T cells with 3-oxoLCA treatment for 12h (n=3). F. Western blot assay for LC3B, p62 and Pink1 protein expression in cultured mouse CD4^+^ T cells with 3-oxoLCA treatment for 24h (n=3). G. Transmission electron microscopy (TEM) showing the mitochondrial morphology of Tfh cells treated with or without 3-oxoLCA (n=4). Scale bars: 1 μm. Mitochondrial area was calculated. The graphs show the data as mean ± SEM. *p < 0.05, **p < 0.01, ***p < 0.001, ****p < 0.0001.

To elucidate the mechanism underlying 3-oxoLCA-mediated repression of Tfh cells differentiation, we performed RNA sequencing (RNA-seq) on naive mouse CD4⁺ T cells cultured under Tfh-polarizing conditions with or without 3-oxoLCA treatment (Figure 4A). Comparative analysis identified 428 differentially expressed genes (287 upregulated and 141 downregulated) in response to 3-oxoLCA, using thresholds of a ≥2-fold change and an adjusted p-value < 0.05 (Figure 4B). This transcriptomic profile confirmed the downregulation of key Tfh cell-related genes (Figure S3C). Subsequent gene set enrichment analysis (GSEA) revealed significant enrichment of pathways involved in mitophagy, autophagy, and rheumatoid arthritis among the genes altered by 3-oxoLCA (Figure 4C). A heatmap visualization further demonstrated the coordinated upregulation of multiple genes within the mitophagy pathway (Figure 4D). Consistent with the RNA-seq data, quantitative RT-PCR analysis confirmed that 3-oxoLCA treatment significantly elevated the mRNA levels of several core mitophagy-related genes, including *Atg5*, *Atg7*, *Ulk1*, *Becn1*, *Pink1*, *Bnip3*, and *Fundc1* (Figure 4E). At the protein level, LC3II/LC3I, Pink1 and p62 are key mediators of mitophagy (13. 3-oxoLCA increased the LC3-II/LC3-I ratio and Pink1 expression, while decreasing p62 levels—a pattern indicative of enhanced mitophagic flux (Figure 4F). Direct observation by transmission electron microscopy (TEM) corroborated these findings, showing a marked reduction in mitochondrial area within 3-oxoLCA-treated CD4⁺ T cells (Figure 4G). Collectively, these data demonstrate that 3-oxoLCA promotes intracellular mitophagy in differentiating Tfh cells.

**Figure 5:**
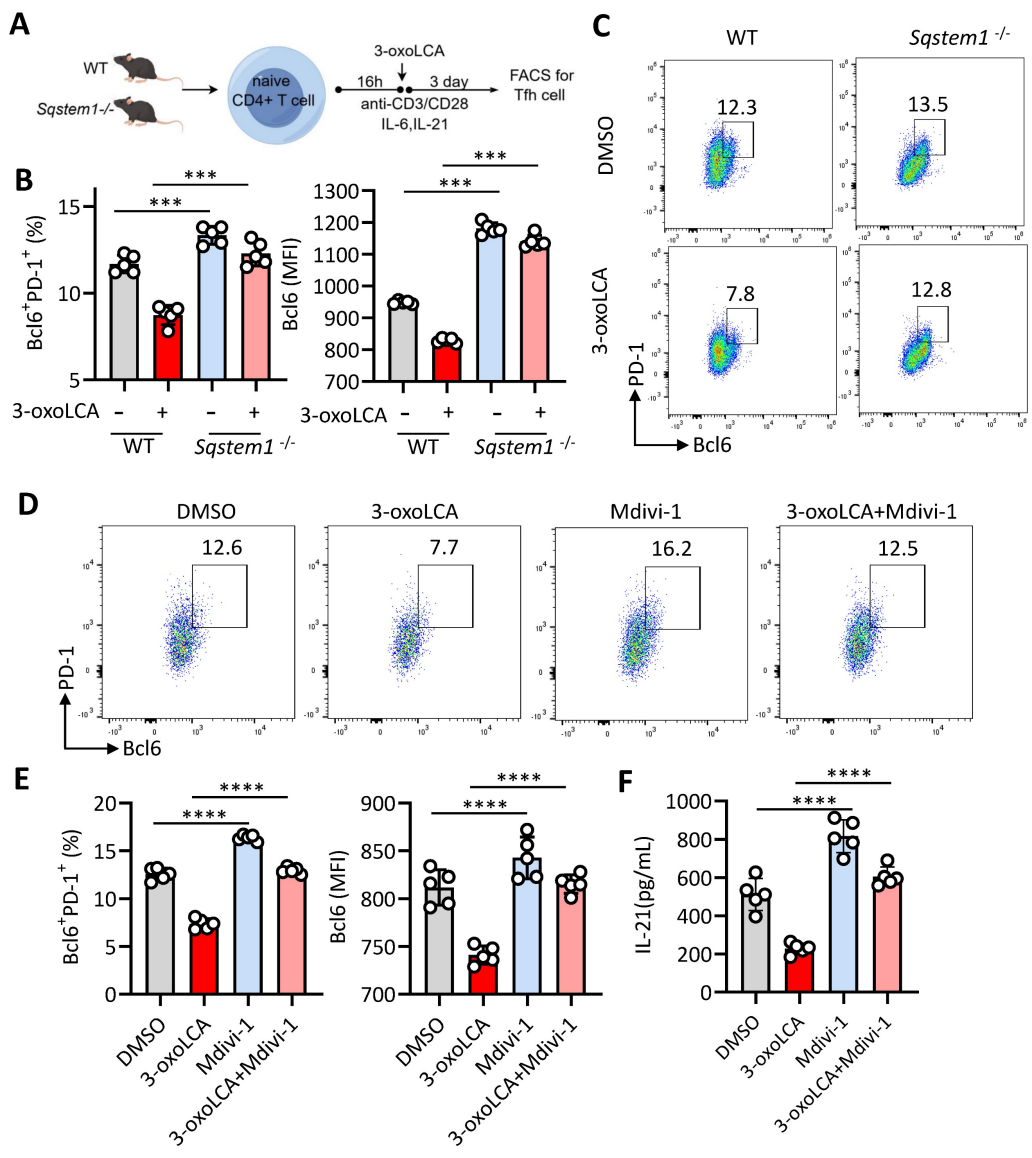
** Mitophagy inhibition restores 3-oxoLCA-ristricted Tfh cells response in culture.** A. Workflow of the experimental procedure of naïve CD4^+^ T cells from WT C57BL/6 mice and *Sqstem1*^-/-^ mice, activation with anti-CD3/CD28 and 3-oxoLCA treatment. B-C. Analysis of PD-1^+^Bcl6^+^ Tfh cells in cultured naïve CD4^+^ T cells from WT and *Sqstem1*^-/-^ mice (n=5). D-F. Representative FACS plots (D) and statistics of PD-1^+^Bcl6^+^ Tfh cells (E), and ELISA analysis of IL-21 concentrations (F) in cultured naïve CD4^+^ T cells from WT C57BL/6 mice with anti-CD3/CD28 activation, followed by treatment with Mdivi-1, plus 3-oxoLCA treatment for 2 days (n=5). The graphs show the data as mean ± SEM. *p < 0.05, **p < 0.01, ***p < 0.001, ****p < 0.0001.

Of note, *Sqstem1*, encoding p62 and play an important role in mitophagy 14, the transcripts expression was significantly upregulated in our RNA-seq data (Figure 4D). Therefore, we inducted *Sqstem1*^-/-^ mice for further investigation. Naïve CD4^+^ T cells from WT C57BL/6 mice and *Sqstem1*^-/-^mice were stimulated with anti-CD3/CD28 plus 3-oxoLCA treatment (Figure 5A). CD4^+^ T cells deficient in *Sqstem1* exhibited a marked increase in their ability to generate Tfh cells relative to WT controls. An inhibitory effect of 3-oxoLCA on Tfh cells frequency was still observed in *Sqstem1*^-/-^ CD4^+^ T cells; nevertheless, the frequency was higher than that in treated WT cells. (Figure 5B, C). We next examined the regulatory role of mitophagy modulators on Tfh cells differentiation. To this end, Mdivi-1, a classic inhibitor of mitochondrial division and mitophagy, was applied to activated mouse CD4^+^ T cells. Mdivi-1 treatment increased the proportion of Tfh cells in a dose-dependent manner Figure S4A,B). Moreover, the production of IL-21, along with the mRNA expressions of *Bcl6*, *Cxcr5*, *Il21*, *Pdcd1*, *Icos* and *Cd40l* were increased with Mdivi-1treatment (Figure S4C,D). Carbonylcyanide 3-chlorophenylhydrazone (CCCP), a classic activator of mitophagy, strongly decreased the proportion of Tfh cells dose dependently (Figure S4E,F), as well as reduced IL-21 secretion and Tfh cell related gene (*Bcl6*, *Cxcr5*, *Il21*, *Pdcd1*, *Icos* and *Cd40l*) expressions (Figure S4G, H). These data supported that mitophagy modulates Tfh cell generation and IL-21 secretion. We speculated that pretreated with Mdivi-1 could effectively reverse the inhibiting effect of 3-oxoLCA on Tfh cells. Mdivi-1 treatment not only increased the baseline Tfh cell ratio but also rescued the 3-oxoLCA-induced reduction in Tfh cells percentage in WT CD4^+^ T cells (Figure 5D). The expression of Bcl6 protein (Figure 5E), IL-21 secretion (Figure 5F), as well as Tfh cell signature genes (*Bcl6*, *Cxcr5*, *Il21*, *Pdcd1*, *Icos* and *Cd40l*) were markedly enhanced in 3-oxoLCA combined with Mdivi-1 treatment compared to 3-oxoLCA alone (Figure S5A). Furthermore, we performed the experimental of WT or *Sqstem1*^-/-^ C57BL/6 mice with NP-OVA/CFA immunization, with or without 3-oxoLCA (Figure S5B). Unexpectedly, *Sqstem1*^-/-^ mice immunized with NP-OVA/CFA generated higher titers of NP-specific IgGs compared to WT controls. And the inhibitory effect of 3-oxoLCA on the antibody response was significantly attenuated in *Sqstem1*^-/-^ mice. While 3-oxoLCA effectively suppressed IgGs levels in WT mice, its suppressive effect was markedly reduced or absent in the *Sqstem1*^-/-^ background (Figure S5C).

Indeed, previous studies have unveiled the critical role of mTOR signaling pathway, especially the mTORC1, in the regulation of autophagy and mitophagy (13. In addition, mTORC1 also closely involved in the development, differentiation, and function of Tfh cells 15, 16. Thus, we further investigated whether 3-oxoLCA regulated mTOR pathway in Tfh cells. Pursuing this, we activated primary murine naïve CD4^+^ T cells from spleen and lymph nodes and then cultured under Tfh polarizing condition *in vitro* in the presence of 3-oxoLCA or DMSO. We observed that the level of phosphorylated-mTOR (p-mTOR)/mTOR, p-p70S6K/p70S6K, and p-S6/S6 were all declined, indicating that the mTOR signaling was inhibited Figure S6. Previous studies reported that mTORC1 signaling regulates Tfh cells differentiation through STAT3 signaling, and bile acids also play a role in STAT3 pathway 16, 17. Therefore, we also detected the status of STAT3 signaling pathway under 3-oxoLCA or not. As expected, 3-oxoLCA treatment also induced the decrease of phosphorylated-STAT3/STAT3 Figure S6. Collectively, these observations demonstrate that 3-oxoLCA leads to dysregulated intracellular mitophagy in Tfh cells differentiation, and mitophagy inhibition effectively rescues Tfh cell generation.

### Tfh cell-targeted nanoparticles with Mdivi-1 rescues 3-oxoLCA-induced Tfh cell deficiency

**Figure 6:**
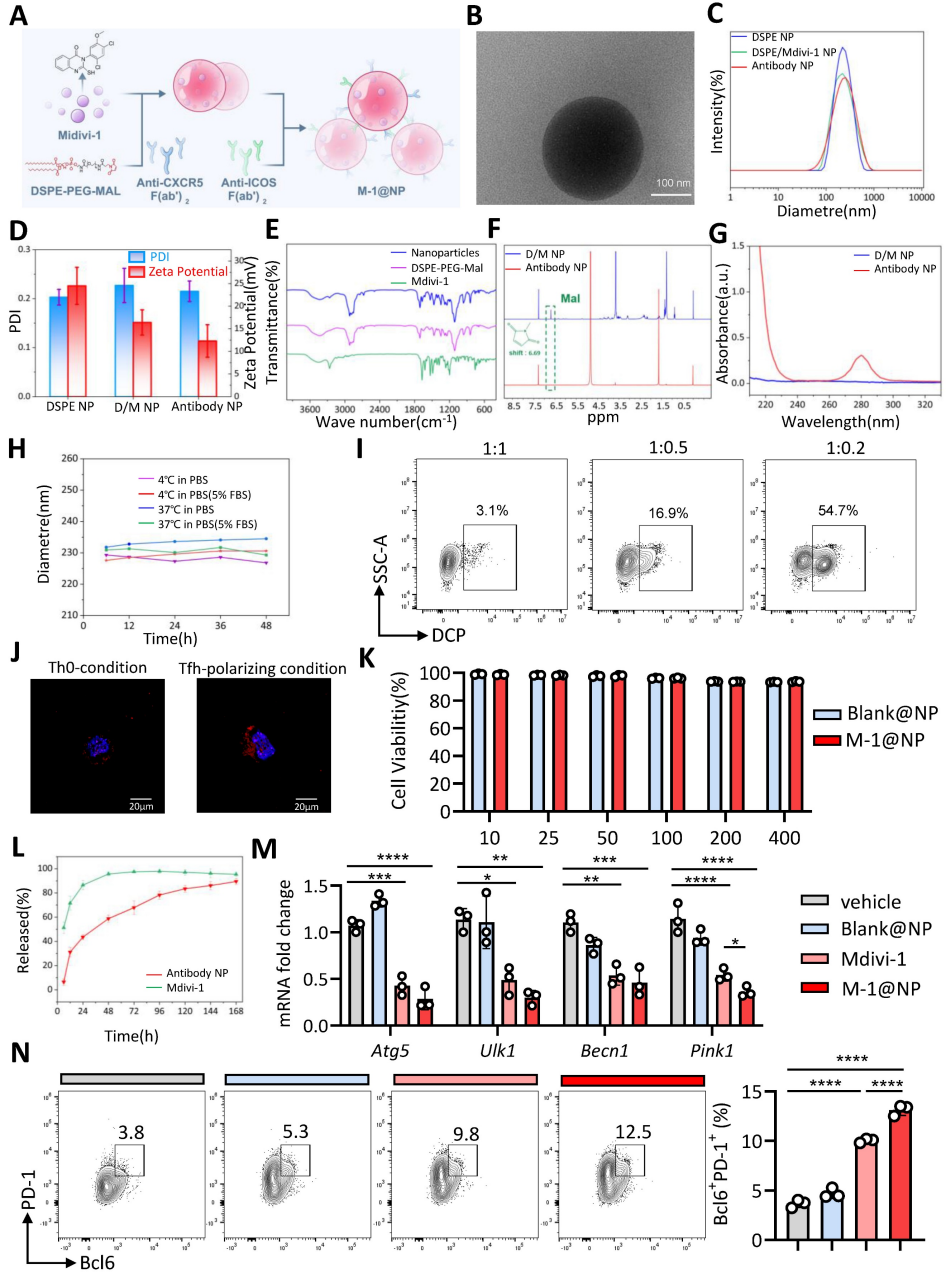
** Targeted Tfh cells nanoparticles with Mdivi-1 rescues 3-oxoLCA-induced Tfh cells deficiency.** A. Schematic strategy for M-1@NP with Mdivi-1, DSPE-PEG, anti-CXCR5 and anti-ICOS by layer-by-layer coating procedure. B. Transmission electron microscopy (TEM) showing the morphology imaging of the structure of M-1@NP. C. Dynamic light scattering (DLS) measurements before and after antibody conjugation. D. Hydrodynamic diameter and Zeta potential of the nanoparticles. E. Fourier transform infrared spectroscopy confirmed the chemical modification. F. Nuclear magnetic resonance spectroscopy showed peaks associated with functional groups, including maleimide (Mal), which shifted after antibody coupling. G. UV-VIS absorption spectroscopy of nanoparticles. H. The diameter of M-1@NP at body temperature or low temperature. I. FACS analysis showing fluorescence intensity of M-1@NP of different ratios of anti-CXCR5 and anti-ICOS. J. Immunofluorescence staining showing fluorescence intensity of M-1@NP in mouse CD4^+^ T cells under Th0-condition and Tfh-polarizing condition. K. Cell viability assay (CCK-8) assessing the cytotoxicity of Black@NP and M-1@NP on Tfh cells (n=3). L. *In vitro* release profile of M-1@NP compared to free Mdivi-1. M. Analysis of Tfh-related genes in mouse naïve CD4^+^ T cells with Black@NP, Mdivi-1 and M-1@NP for an additional 12h by rt-qPCR (n=3). N. Representative FACS plots and statistics of Bcl6^+^PD-1^+^ Tfh cells after treatment for 3days (n=3). The graphs show the data as mean ± SEM. *p < 0.05, **p < 0.01, ***p < 0.001, ****p < 0.0001.

Given the striking role of Mdivi-1 on Tfh cell differentiation *in vitro*, we proposed that Mdivi-1 could also promotes Tfh cells differentiation and improve 3-oxoLCA-induced impair GC reactions *in vivo*. Since Mdivi-1 is a fat-soluble compound and widely distributed *in vivo* with a very short half-life and low bioavailability 18. In order to enhance the proportion and function of Tfh cells, we constructed nanoparticles based on DSPE-PEG, with surface-conjugated anti-CXCR5 and anti-ICOS Fab fragments, and loaded Mdivi-1 inside the nanoparticles to form the nano-adjuvant M-1@NP, enabling targeted delivery of Mdivi-1 to Tfh cells (Figure 6A). Under transmission electron microscopy, the shape and structure of M-1@NP are spherical and evenly distributed (Figure 6B). The polydispersity index (PDI) values were relatively low in all groups, and the diameter of the nanoparticles were 100-150nm. And the hydromechanical diameter of the nanoparticles increased slightly after conjugation, while the Zeta potential significantly decreased (Figure 6C,6D). Fourier transform infrared spectroscopy (FTIR) confirmed the chemical modification, indicating that the drug package was successfully set, and DSPE-PEG-Mal was very similar to the nanoparticle profile, with no chemical changes during loading (Figure 6E). Nuclear magnetic resonance (NMR) spectroscopy showed peaks associated with functional groups, including maleimide (Mal), which shifted after antibody coupling, confirming the chemical binding of the antibody to the nanoparticle surface (Figure 6F). In the UV-VIS absorption spectrum, M-1@NP shows peaks associated with antibodies indicating successful functionalization (Figure 6G). M-1@NP is generally stable at body temperature or low temperature, with or without 5% FBS (Figure 6H). *In vitro*, the load rate of Mdivi-1 in M-1@NP was 7.26% though high performance liquid chromatography (HPLC) assay, and the release rate of Mdivi-1 from M-1@NP was over 90% within 7 days (Figure 6L). M-1@NP had the highest targeting rate to Tfh cells when the ratio of anti-ICOS: anti-CXCR5 was 1:0.2 (Figure 6I). After treating with M-1@NP labeled with rhodamine, the fluorescence intensity of CD4^+^ T cells under Tfh-polarzing condition were drastically higher than T cells under Th0-condition, indicating that the targeting ability of M-1@NP to Tfh cells was significantly enhanced (Figure 6J). Furthermore, we also systemically evaluated the cellular uptake of fluorescently labeled nanoparticles across key immune cell subsets in the spleen after *in vivo* administration. Results shown that the mean fluorescence intensity (MFI) of the nanoparticle signal was significantly higher in Tfh cells (CD4^+^CXCR5^+^BCL6^+^) compared to all other examined populations, including DCs, macrophages, CD8^+^ T cells, non-Tfh CD4^+^ T cells and B cells Figure S7.

Biosafety is the basic application condition for drug applications. We observed the nanoparticles did not show significant cytotoxicity to T cell from WT mice within 400 µg/mL by CCK-8 assay (Figure 6K). Moreover, the HE staining indicated there were no significant morphologic changes at the experimental dose in main organs including heart, lung, liver, spleen, and kidney Figure S8A), and the level of ALT and AST remain normal after 12 days (Figure S8B). The above results indicate that M-1@NP were safe and suitable for biological applications. Then, we found that CD4^+^ T cells treated with M-1@NP significantly promoted the proportion of Bcl6^+^PD-1^+^ cells compared to Mdivi-1 alone (Figure 6N), as well as the mRNA expression of Tfh cells relative genes (Figure 6M). Taken together, the designed nanoparticles exhibit excellent Tfh-target effects and could drastically increase Tfh cells proportion *in vitro*.

### M-1@NP improved 3-oxoLCA induced Tfh cells deficiency and GC response *in vivo*

**Figure 7:**
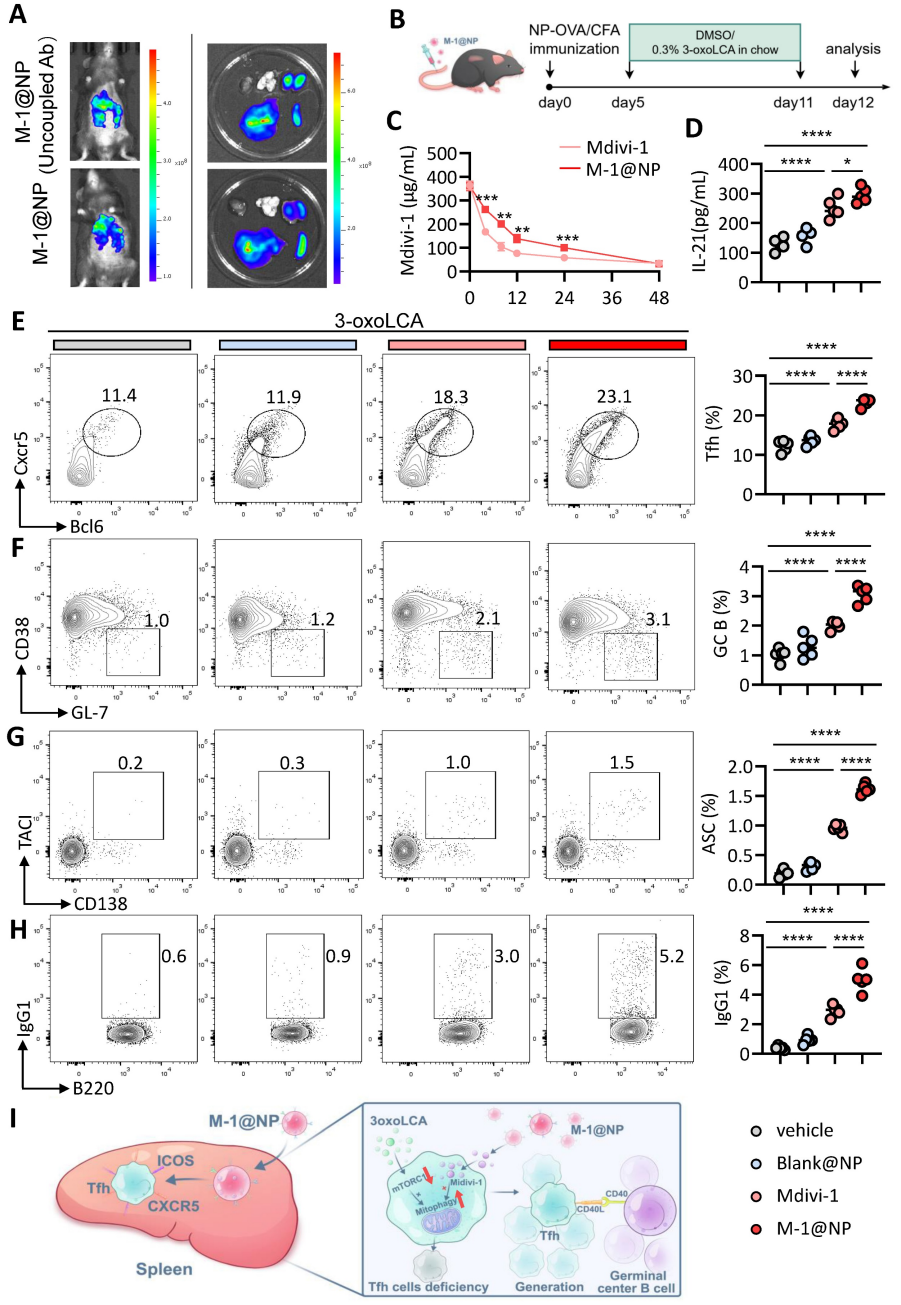
** Tfh cell-targeted delivery of M-1@NP compensates for 3-oxoLCA-induced suppression of Tfh cells responses *in vivo*.** A. *In vivo* and *ex vivo* fluorescence imaging and semiquantitative analysis of M-1@NP distribution in mice organs after tail vein injection. B. Schematic diagram of the experimental schedule. C. HPLC analysis of plasma concentration of Mdivi-1 at different time points in mice blood (n=3). D. ELISA measurement of serum IL-21 concentration in mice with treatment (n=5). E-H. Analysis of Tfh (CD44⁺Foxp3⁻ CD4⁺ T cells) (E), GC B cells (F), ASCs (G), and IgG1⁺ cells (H) in murine spleen by FACS (n=5). I. Schematic representation showing how M-1@NP inhibits mitophagy and promotes Tfh cells response. The graphs show the data as mean ± SEM. *p < 0.05, **p < 0.01, ***p < 0.001, ****p < 0.0001.

We further examined the efficiency of M-1@NP in Tfh cells and GC response *in vivo*. Firstly, we evaluated the target efficiency of M-1@NP to Tfh cells in mice, we observed that the fluorescence intensity was stronger in spleen in mice injected with M-1@NP, while the mice treated with M-1@NP (Uncoupled Ab) exhibit higher fluorescence intensity in liver (Figure 7A). HPLC results shown that 80% of free Mdivi-1 were rapidly cleared within 8 h, while M-1@NP was continuously released with a slow decrease in blood (Figure 7C). Then, we examined the efficiency of M-1@NP in restoring Tfh cells and GC response in immunized mice model. To this end, we treated 3-oxoLCA-fed mice with PBS, Black@NP, Mdivi-1 and M-1@NP respectively (Figure 7B). Firstly, we evaluated the concentration of serum IL-21 in mice, results shown that mice treated with Mdivi-1 have higher levels of IL-21 in serum compare to PBS and Black@NP mice, and M-1@NP treated mice also have higher IL-21 levels compare to or Mdivi-1treated alone (Figure 7D). Then, as depicted in Fig. 7E-G, we observed a significant increase in the frequencies of Tfh cells, GC B cells, and plasma cells in spleen of M-1@NP and Mdivi-1- treated mice compared with the mice treated with PBS and Black@NP, and the M-1@NP had a high proportion of Tfh, GC B celss and ASCs, as well as the production of IgG1 in B220^+^ cells than Mdivi-1 group (Figure 7H). Collectively, treatment with M-1@NP significantly restored Tfh cells differentiation in 3-oxoLCA accumulated mice, which led to enhanced GC reactions *in vivo* (Figure 7I).

## Discussion

This study explores the impact of bile acids on Tfh cells differentiation and vaccine-induced immunity in both murine and human systems. We found that 3-oxoLCA suppresses Tfh cells differentiation and IL-21 production *in vitro* and diminishes vaccine responses *in vivo*. Mechanistically, this effect is underpinned by the induction of mitophagy. Furthermore, we developed a nanoparticle-based delivery system loaded with a mitophagy inhibitor to target Tfh cells, which successfully reversed the impaired germinal center response associated with 3-oxoLCA treatment.

The involvement of bile acids in vaccine response was hinted at by previous investigation. Alexander *et al*. reported that high levels of succinate, phenylalanine, taurolithocholate and taurodeoxycholate in faeces were all associated with poorer COVID-19 vaccine-induced antibody response in inflammation bowel disease patients (19. Another study also demonstrated that serum metabolomics analysis of antibiotic-induced defective vaccine response focus on bile acids 20. In animal model, BDL-induced liver injury leads to impaired T-cell responses to vaccination and viral infection in mice, subsequently leading to persistent infection 21. We also previously reported that serum bile acids negatively related to the titer of HBV surface antibody in BA children 22. These evidences all indicated that bile acids might regulate vaccine response and the immune cells related to vaccine response. In the current investigation, we unveiled the immunomodulating effect of 3-oxoLCA on Tfh cells, and provided an explanation for the role of bile acids in vaccine response.

Several studies have demonstrated that bile acids exert immunomodulating effects on macrophages, dendritic cells, monocytes, and T cells 3-10. For example, bile acids are essential to maintain a tolerogenic phenotype of macrophage via the BA receptor TGR5 through blocking NLRP3 inflammasome-dependent pathway 23. In dendritic cells exposed to LPS, DCA suppresses the expression of pro-inflammatory markers like IL-6 24. Apart from innate immunity, bile acids modulate the differentiation and function of T cells. Previous studies have revealed that 3-oxoLCA and isoLCA both restrain Th17 cell differentiation by directly blocking the transcription of the nuclear hormone receptor (RORγt), in the meanwhile, isoalloLCA exhibit inhibitory effect on Treg cells through mitoROS-Foxp3 axis 4, 5. Moreover, NorUDCA regulates CD8^+^ T cells by targeting mTORC1 and intracellular metabolism, resulting in remission of hepatic inflammation 10. Of note, in this study, we unveiled that 3-oxoLCA inhibits Tfh cell differentiation and function though enhanced mitophagy. Our research enriches understanding of the immunomodulating effect of bile acids on CD4^+^ T cells.

Mechanically, we discovered mitophagy involved in the regulating of 3-oxoLCA in Tfh cells differentiation and function. Mitophagy is an intracellular selective autophagy process that specifically recognizes and degrades damaged or dysfunctional mitochondria to maintain a stable intracellular environment. This process critically regulates cellular energy metabolism, maintains proteostatic control, and contributes to disease pathogenesis. Mitophagy is activated when mitochondrial damage exceeds the capabilities of other quantity and quality control methods, or when mitochondria are removed for cellular metabolic purposes 25. Recently, emerging lines of evidence indicates that mitophagy is directly engaged in the development and differentiation of immune cells including T cells, natural killer (NK) cells and macrophages 26-33. For instance, previous study discovered that metformin improves autophagy and mitochondrial function of Th17 cells and largely in parallel to ameliorate a newly defined inflammaging profile that echoes inflammation in diabetes 28. Moreover, Kathleen *et al.* reported that naïve CD4^+^ and CD8^+^ T cells from *Parkin*-deficient mice exhibit enhanced activation marker expression and proliferative responses to alloantigen, and promoting process of the skin-graft rejection 34. In the current study, we first reported that enhanced mitophagy restricted the differentiation of Tfh cells and the antibody response *in vitro* and *in vivo*. We found that Mdivi-1 could significantly increase the proportion of cultured Tfh cells and the function of IL-21 secretion, whereas activation of mitophagy, using CCCP could strongly restrained the Tfh cells differentiation.

The design and fabrication of nanostructured materials present significant potential for creating novel immunomodulatory agents, since these nano-scale systems enable enhanced manipulation and targeted delivery of immunologically active substances to specific locations 35. The targeted administration of immunotherapeutic agents plays a key role in diverse medical areas, including oncology, autoimmune disorders, and organ transplantation 36-38. In this study, we fabricated a codelivery system termed M-1@NP, which is specifically directed at Tfh cells within the spleen and lymph nodes. This system functions to suppress mitophagy in Tfh cells, thereby enhancing their cellular response. Using anti-CXCR5 and anti-ICOS-coated nanoparticles and Mdivi-1, we demonstrate that effective vaccine response via promoting Tfh cells. Compared with vaccination with Mdivi-1 alone, mice vaccinated with the M-1@NP showed superior GC response after immunization. The insights from this study may contribute to the development of new methods for boosting vaccine efficacy, shedding light on applications for people with high serum bile acids.

The strengths of this work include the confirmation of our findings in 3-oxoLCA induced Tfh cells deficiency in cultured Tfh cells and immunized mice and the role of mitophagy in Tfh cells biology. Tfh cell-targeted nanoparticles present considerable novelty in this field. The main limitation of our study is that we did not investigate the potential correlation between Tfh cells and 3-oxoLCA in either healthy individuals or patients with cholestatic liver disorders. Further studies are warranted to examine the association of Tfh cells with 3-oxoLCA or other bile acid species in people. Additionally, the biosafety profile of the studied nanoparticles necessitates further verification through larger-scale *in vivo* studies.

In summary, this present study revealed that 3-oxoLCA impairs the differentiation and function of Tfh cells by promoting intracellular mitophagy, and Tfh-targeted nanoparticles could effectively improve Tfh cells proportion and GC response. Our findings not only reveal the pathway that specifically controls the survival of Tfh cells but also provide a strategy to boost Tfh cells function and improve protective humoral immunity in vaccination.

## Supplementary Material

Supplementary figures and tables.

## Data Availability

The datasets generated and/or analysed during the current study are not publicly available due [REASON WHY DATA ARE NOT PUBLIC] but are available from the corresponding author on reasonable request.

## Abbreviations

Tfh: Follicular helper T cells
GC: Germinal center
ASC: Antibody secreting cell
Bcl6: B-cell lymphoma 6 protein
Cxcr5: C-X-C chemokine receptor type 5
IL-21: Interleukin 21
ICOS: Inducible costimulatory
PD-1: Programmed death1
BA: Biliary atresia
GSEA: Gene Set Enrichment Analysis
RNA-seq: RNA sequencing
KEGG: Kyoto Encyclopedia of Genes and Genomes
GO: Gene Ontology
