# Supplementary material for: Engineering Tfh-Specific Nanoadjuvant to Counteract Bile Acid-Induced Mitophagy and Vaccine Hyporesponsivenes
Source: Theranostics. 2026 Mar 30;16(10):5741–58. doi: 10.7150/thno.125668 (PMC13081164; doi:10.7150/thno.125668)
Supplement: Supplementary file 1 — Supplementary figures and tables. [file thnov16p5741s1.pdf]

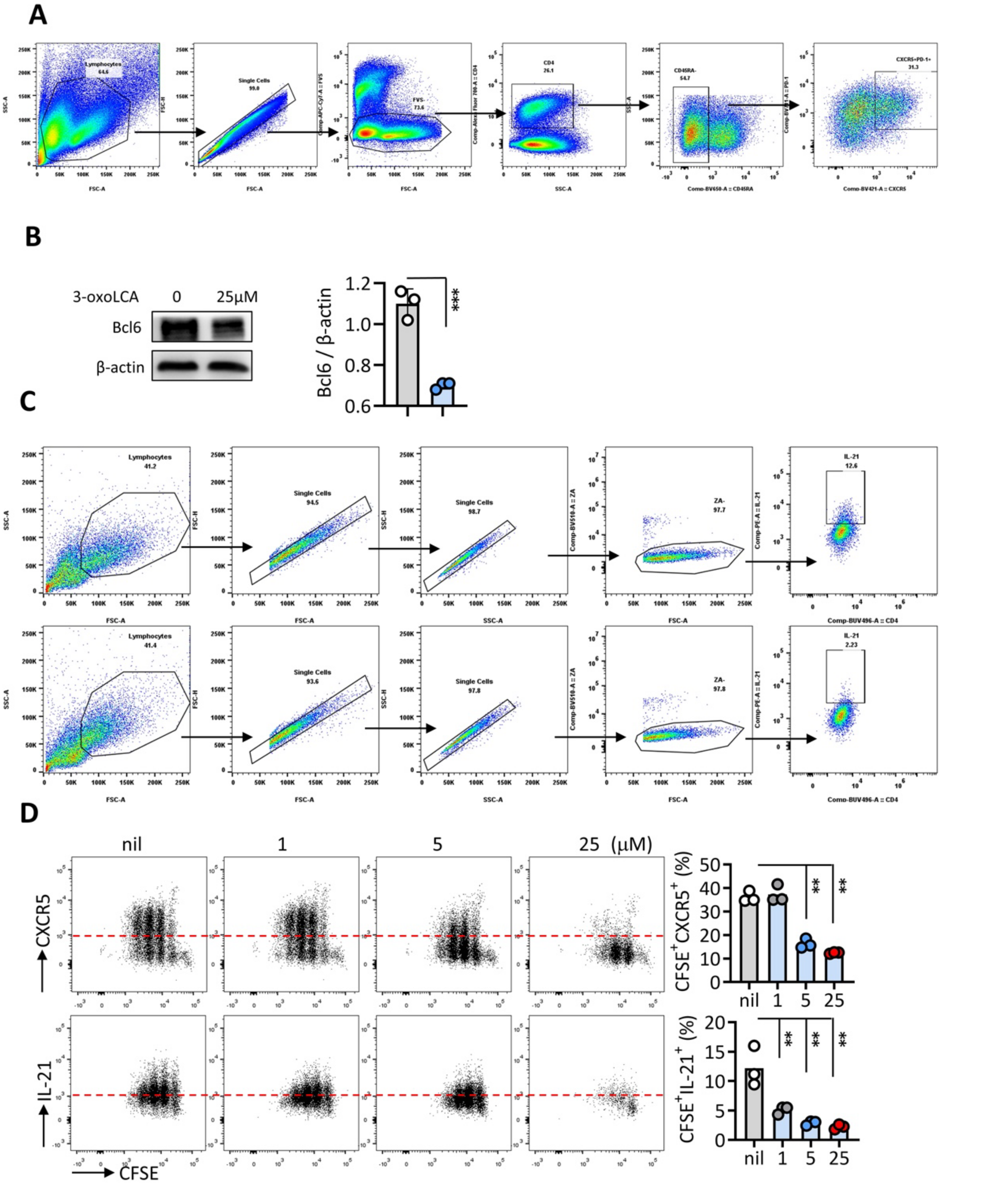


**Figure S1. 3-oxoLCA affect Tfh cells differentiation in human CD4$^{+}$ T cells.**

A. The gating strategy to analyse Tfh cell subsets in cultured human CD4$^{+}$ T cells.

B. Bcl6 expression in WT mouse naive CD4$^{+}$ T cells stimulated with 3-oxoLCA by Western blot (n=3).

C. The gating strategy to analyse IL-21 positive cells in cultured human CD4$^{+}$ T cells.

D. The proliferation of cultured human CFSE$^{+}$CXCR5$^{+}$ and CFSE$^{+}$IL21$^{+}$ T cells by CFSE assay (n=3).

The graphs show the data as mean ± SEM. *$p < 0.05$, **$p < 0.01$, ***$p < 0.001$, ****$p < 0.0001$.

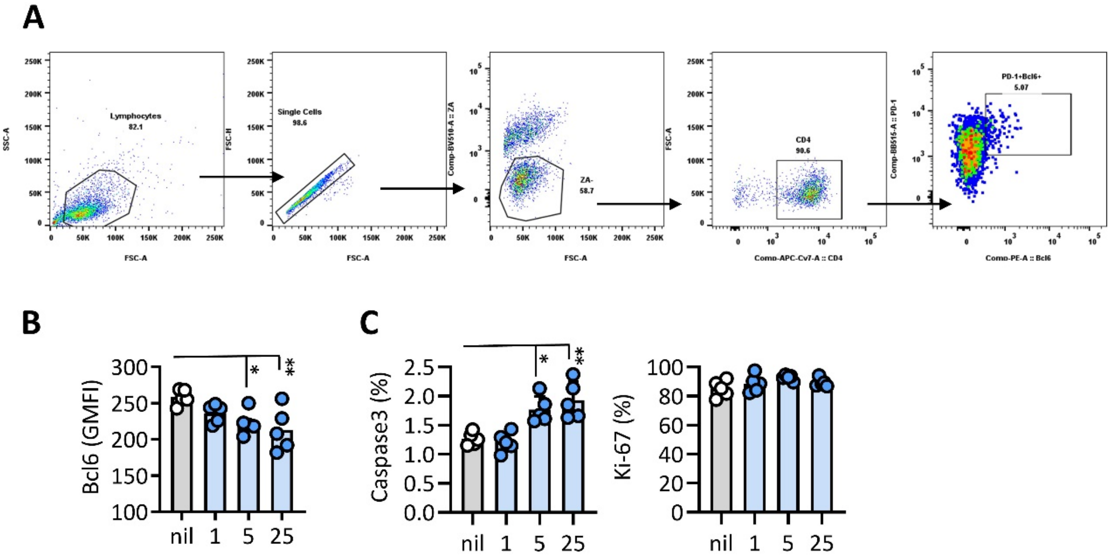


**Figure S2. 3-oxoLCA affect Tfh cells differentiation in mouse CD4$^+$ T cells.**

A. The gating strategy to analyse Tfh cell subsets in cultured mouse CD4$^+$ T cells.

B. The geometric mean fluorescence intensity (GMFI) of Bcl6 expression at different concentrations of 3-oxoLCA (n=5).

C. The percentage of Caspase-3 and Ki-67 of CD4$^+$ T cells at different concentrations of 3-oxoLCA (n=5).

The graphs show the data as mean ± SEM. *$p < 0.05$, **$p < 0.01$, ***$p < 0.001$, ****$p < 0.0001$.

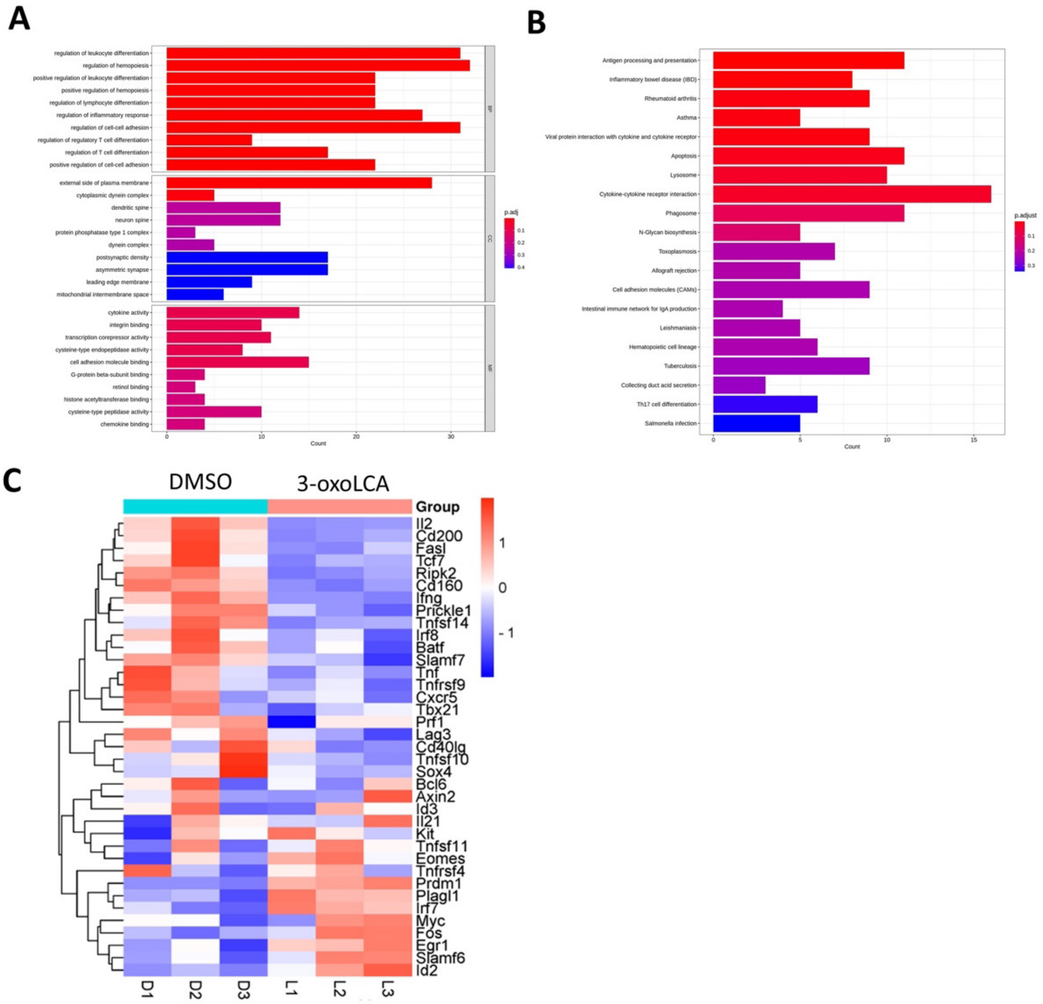


**Figure S3. 3-oxoLCA inhibit Tfh cells through improve mitophagy.**

A. Gene ontology enrichment analysis was performed on the DEGs that were differentially regulated by either 3-oxoLCA resulted in changes in the expression of genes involved in several biological processes.

B. Top enriched pathways of DEGs in 3-oxoLCA treated Tfh cells by Kyoto Encyclopedia of Genes and Genomes (KEGG) analysis.

C. Heatmap showing the RNA-seq analysis of Tfh-related genes in mouse naïve CD4$^{+}$ T cells with anti-CD3/CD28 activation for 12h, and further treated with 3-oxoLCA or DMSO for 12 h.

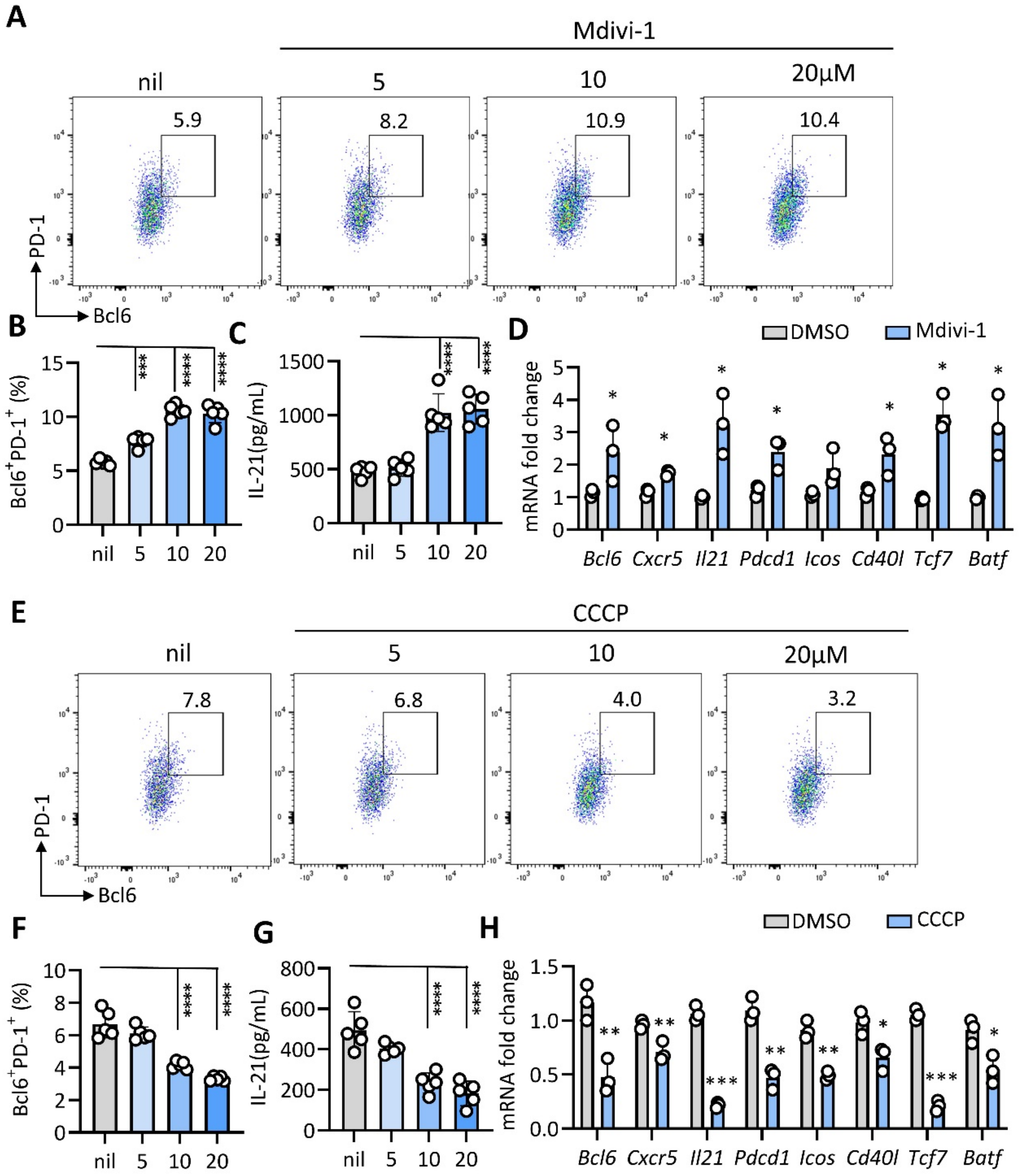


**Figure S4. Modulation of intercellular mitophagy regulates Tfh cells differentiation.**

A-B. Analysis of PD-1$^{+}$Bcl6$^{+}$ Tfh cells in cultured naïve CD4$^{+}$ T cells from WT mice with mitophagy inhibitor (Mdivi-1) for 3days (n=5).

C. ELISA analysis of IL-21 production in naïve CD4$^{+}$ T cells from WT mice with Mdivi-1 for 3days (n=5).

D. Tfh-related genes in naïve CD4$^{+}$ T cells from WT mice treated with Mdivi-1 for 12h by real-time PCR (n=3).

E-F. Analysis of PD-1$^{+}$Bcl6$^{+}$ Tfh cells in cultured naïve CD4$^{+}$ T cells from WT mice with mitophagy inducer (CCCP) for 3days (n=5).

G. ELISA analysis of IL-21 production in naïve CD4$^{+}$ T cells from WT mice with mitophagy inducer (CCCP) for 3days (n=5).

H. Tfh-related genes in naïve CD4$^{+}$ T cells from WT mice treated with CCCP for 12h by real-time PCR (n=5).

The graphs show the data as mean ± SEM. *$p < 0.05$, **$p < 0.01$, ***$p < 0.001$, ****$p < 0.0001$.

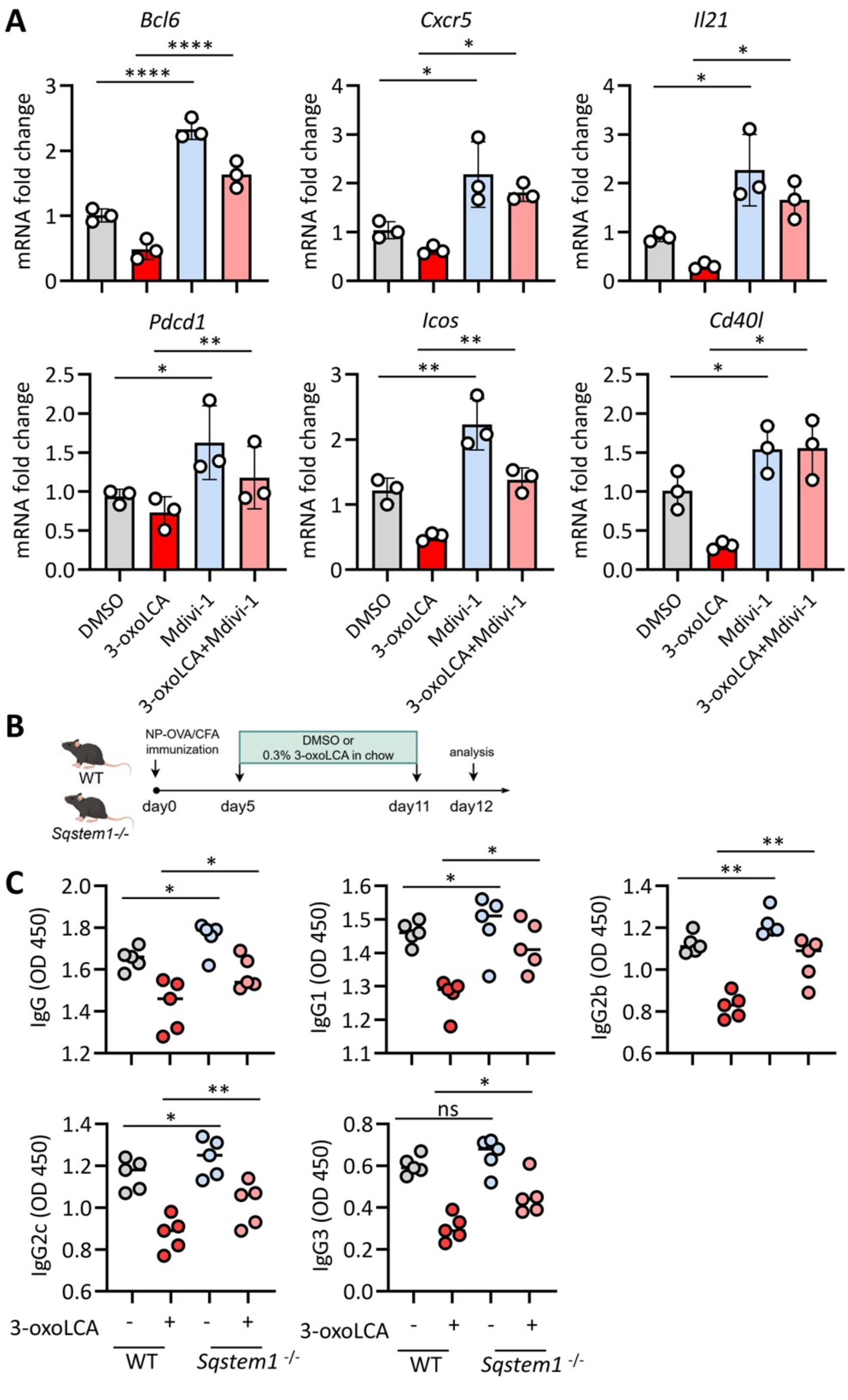


**Figure S5. Inhibition of mitophagy restores 3-oxoLCA induced Tfh cells differentiation deficiency.**

A. Real-time quantitative PCR analysis of mRNA abundance of Tfh cell-related genes in DMSO, 3-oxoLCA, Mdivi-1 and 3-oxoLCA + Mdivi-1 group (n=3).

B. Workflow of the experimental procedure of WT or *Sqstem1*$^{-/-}$ C57BL/6 mice with NP-OVA/CFA immunization (subcutaneous), 3-oxoLCA (oral gavage) and analysis.

C.ELISA assay for serum NP-specific IgG1, IgG2b, IgG2c and IgG3 titers from in WT or *Sqstem1*$^{-/-}$ C57BL/6 mice immunized with NP-OVA/CFA, followed by 3-oxoLCA treatment (n=5).

The graphs show the data as mean ± SEM. *$p < 0.05$, **$p < 0.01$, ***$p < 0.001$, ****$p < 0.0001$.

**Figure S6. 3-oxoLCA3-oxoLCA reduces Tfh cells blasting associated with attenuated mTORC1 and STAT3 signaling pathway.**

The phosphorylation of key signaling proteins (mTOR, p70S6K, S6, STAT3) in WT naïve $CD4^+$ T cells was compared by Western blot following treatment with vehicle control or 25 μM 3-oxoLCA (n=3).

The graphs show the data as mean ± SEM. *$p < 0.05$, **$p < 0.01$, ***$p < 0.001$, ****$p < 0.0001$.

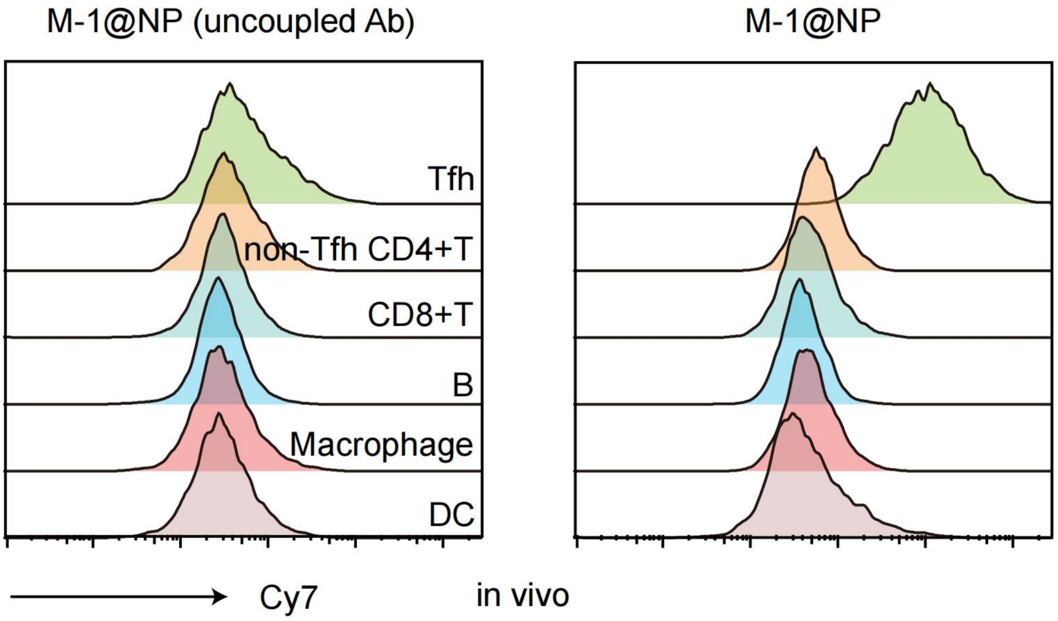


**Figure S7. The distribution of M-1@NP in different lymphocyte subsets in vivo by flow cytometry.**

The histogram of flow cytometry showing fluorescence intensity of M-1@NP in different immune cell subpopulations in mouse spleen.

The graphs show the data as mean ± SEM. **p* < 0.05, ***p* < 0.01, ****p* < 0.001, *****p* < 0.0001.

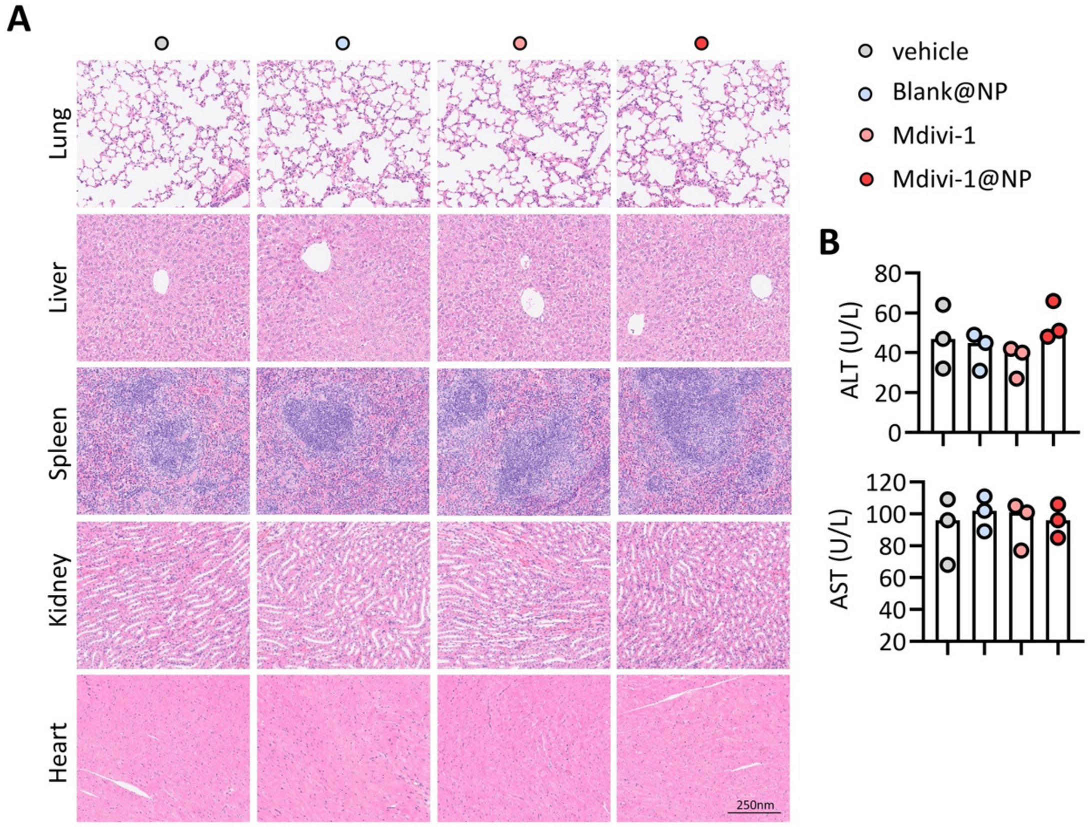


**Figure S8. In vivo biocompatibility assessment.**

A. Major organs of mice were taken for H&E staining at time points of 12 days after M-1@NP injection.

B. Serum glutathione transaminase and ghrelin levels in mice injected with the M-1@NP in the tail vein after 12 days (n = 3).

The graphs show the data as mean ± SEM. *$p < 0.05$, **$p < 0.01$, ***$p < 0.001$, ****$p < 0.0001$.

**Supplementary Table S1.** PCR primers of mouse used in this study

| genes | Forward Sequence | Reverse Sequence |
|---|---|---|
| Bcl6 | CAGAGATGTGCCTCCATACTGC | CTCCTCAGAGAAACGGCAGTCA |
| Cxcr5 | ATCGTCCATGCTGTTCACGCCT | CAACCTTGGCAAAGAGGAGTTCC |
| Il21 | GCCTCCTGATTAGACTTCGTCAC | CAGGCAAAAGCTGCATGCTCAC |
| Pdcd1 | CGGTTTCAAGGCATGGTCATTGG | TCAGAGTGTCGTCCTTGCTTCC |
| Icos | GCAGCTTTCGTTGTGGTACTCC | TGTGTTGACTGCCGCCATGAAC |
| Cd40l | GAACTGTGAGGAGATGAGAAGGC | TGGCTTCGCTTACAACGTGTGC |
| Tcf7 | CCTGCGGATATAGACAGCACTTC | TGTCCAGGTACACCAGATCCCA |
| Batf | CACAGAAAGCCGACACCCTTCA | GCTGCTCAGCACTGATGTGAAG |
| Atg5 | CTTGCATCAAGTTCAGCTCTTCC | AAGTGAGCCTCAACCGCATCCT |
| Atg7 | CCTGTGAGCTTGGATCAAAGGC | GAGCAAGGAGACCAGAACAGTG |
| Ulk1 | GCAGCAAAGACTCCTGTGACAC | CCACTACACAGCAGGCTATCAG |
| Becn1 | CAGCCTCTGAAACTGGACACGA | CTCTCCTGAGTTAGCCTCTTCC |
| Pink1 | CGACAACATCCTTGTGGAGTGG | CATTGCCACCACGCTCTACACT |
| Bnip3 | GCTCCAAGAGTTCTCACTGTGAC | GTTTTTCTCGCCAAAGCTGTGGC |
| Fundc1 | AGACACCACTGGTGGAATCGAG | CCTTCTGGAATAAAAATCCTGCAC |

**Supplementary Table S2.** PCR primers of human used in this study.

| genes | Forward Sequence | Reverse Sequence |
|---|---|---|
| *BCL6* | CATGCAGAGATGTGCCTCCACA | TCAGAGAAGCGGCAGTCACACT |
| *CXCR5* | TGAAGTTCCGCAGTGACCTGTC | GAGGTGGCATTCTCTGACTCAG |
| *IL21* | CCAAGGTCAAGATCGCCACATG | TGGAGCTGGCAGAAATTCAGGG |
| *PDCD1* | AAGGCGCAGATCAAAGAGAGCC | CAACCACCAGGGTTTGGAACTG |
| *ICOS* | CCCATAGGATGTGCAGCCTTTG | GGCTGTGTTCACTGCTCTCATG |
| *CD40L* | GCGGCACATGTCATAAGTGAGG | GTCCTTGTCTTTTAACGGTCAGC |
